# *Herniosina* Roháček: revised concept, two new species, new key and atlas of male and female terminalia (Diptera, Sphaeroceridae)

**DOI:** 10.3897/zookeys.609.9459

**Published:** 2016-08-08

**Authors:** Jindřich Roháček

**Affiliations:** 1 Silesian Museum, Nádražní okruh 31, CZ-746 01 Opava, Czech Republic

**Keywords:** Diptera, Sphaeroceridae, Herniosina Roháček, 2 new species, key, terminalia, taxonomy, relationships, biology, distribution, W. Palaearctic

## Abstract

The taxonomic concept of *Herniosina* Roháček, 1983 (Diptera: Sphaeroceridae) is revised on the basis of five W. Palaearctic species, thus excluding the E. Nearctic *Herniosina voluminosa* Marshall, 1987 whose inclusion caused the paraphyly of the genus. Two new species, *Herniosina erymantha* **sp. n.** (male only, Greece: Peloponnese) and *Herniosina hamata* **sp. n.** (both sexes, Cyprus), are described and illustrated, and the other three species, *Herniosina bequaerti* (Villeneuve, 1917), *Herniosina horrida* (Roháček, 1978) and *Herniosina pollex* Roháček, 1993, are diagnosed with an atlas of their male and female terminalia. The relationships of the redefined genus and of all its species are discussed, and their biology and distribution are reviewed. A new illustrated key to *Herniosina* species is given.

## Introduction

**Figure 1.**
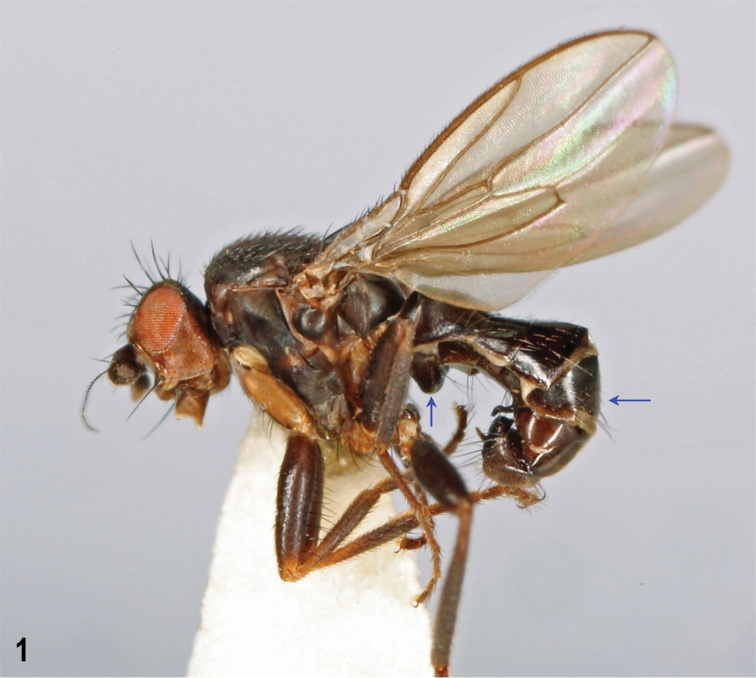
*Herniosina bequaerti* (Villeneuve), male laterally (Czech Republic: Bohemia). Body length cca 2.6 mm. S1+2 and T5 arrowed. Photograph by J. Roháček.

The genus *Herniosina* was established by Roháček (1982, 1983) during the re-classification of the giant assemblage previously included in the genus *Limosina* Macquart, 1835 to comprise two European species of the subfamily Limosininae, viz. *Herniosina bequaerti* (Villeneuve, 1917) and *Herniosina horrida* (Roháček, 1978). The genus was characterized by the peculiar down-curved male abdomen with protruding bulge on abdominal synsternum S1+2 (cf. Fig. 1) and distinctive male genitalia. Discussing its relationships Roháček (1982) suggested it obviously belongs together with the monotypic genera *Limosina* (redefined), *Gigalimosina* Roháček, 1983 and *Apteromyia* Vimmer, 1929 to the *Limosina* genera-group. When describing *Apteromyia newtoni* (a second species of the genus) Marshall and Roháček (1982) hypothesized *Apteromyia* as the closest relative of *Herniosina*. Marshall (1987) described another unusual species of Limosininae and placed it tentatively in *Herniosina* on the basis of (seemingly) similarly protruding male abdominal S1+2 despite his explicit recognition that it was markedly different in other characters of the male genitalia, i. e. lacking all other synapomorphies of the genus as originally delimited. The inclusion of *Herniosina voluminosa* Marshall, 1987 made *Herniosina* a heterogeneous and apparently non-monophyletic group, as was stated already by Roháček (1993) when describing the third European species, *Herniosina pollex* Roháček, 1993. Apart from a record of two unidentified females from Israel (Papp and Roháček 1988: 89, as Herniosina sp. cf. horrida) no further species of *Herniosina* had been known until Roháček (2004) reported about an unnamed species found in Cyprus. However, the latter species has remained undescribed up to the present. Only the recent (2015) discovery of an additional new species in the Peloponnese peninsula initiated the present study which is not only aimed at the descriptions of these two new species but also at revision of taxonomic limits of the genus, its re-definition on the basis of the study of all known species, hypotheses of their relationships as well as at an updated synopsis of their biology and distribution.

## Material and methods

### Material

The material examined is deposited in institutional and private collections as follows:

FSBC
 Faculty of Science, Masaryk University, Brno, Czech Republic 


HNHM
Hungarian Natural History Museum, Budapest, Hungary 


ISNB
Institut Royal des Sciences Naturelles de Belgique, Bruxelles, Belgium 


JRO
 Collection of Dr. Jindřich Roháček, Opava, Czech Republic 


MBP
 Collection of Prof. Miroslav Barták, Praha, Czech Republic 


MMBC
 Moravské zemské muzeum, Brno, Czech Republic 


MNHN
 Entomologie, Muséum National d’Histoire Naturelle, Paris, France 


MSNV
Museo Civico di Storia Naturale, Venezia, Italy 


MZHF
 Universitetets Zoologiska Museum, Helsinki, Finland 


NMPC
 Národní muzeum, Praha, Czech Republic 


PKBS
 Prírodovedecká fakulta Univerzity Komenského, Bratislava, Slovakia 


SMOC
 Slezské zemské muzeum, Opava, Czech Republic 


SMTD
 Staatliches Museum für Tierkunde, Dresden, Germany 


UEBC
 Ústav ekologie lesa, Mendelova zemědělská a lesnická univerzita, Brno, Czech Republic 


VKB
 Collection of Dr. Vladimír Košel, Bratislava, Slovakia 


ZMHB
Museum für Naturkunde der Humboldt-Universität zu Berlin, Berlin, Germany 


### Methods of preparation and study of postabdominal structures

Abdomens of a number of specimens were detached, cleared by boiling several minutes in 10% solution of potassium hydroxide (KOH) in water, then neutralized in 10% solution of acetic acid (CH_3_COOH) in water, washed in water and subsequently transferred to glycerine. Postabdominal structures were dissected and examined in a drop of glycerine under binocular microscopes (Reichert, Olympus). Detailed examinations of genital structures were performed with a compound microscope (JENAVAL). After examination, all dissected parts were put into small plastic tubes containing glycerine, sealed with hot forceps and pinned below the respective specimens.

### Drawing techniques and photography

Legs were drawn on squared paper using a Reichert binocular microscope with an ocular screen. Details of the male and female genitalia were drawn by means of Abbe‘s drawing apparatus on a compound microscope (JENAVAL) at larger magnification (130–500×). Wings were photographed on the same microscope with an attached digital camera (Nikon COOLPIX 4500). Whole specimens were photographed by means of digital camera Canon EOS 5D Mark III with macro lens Canon MP-E 65 mm 1–5× and ring macro flash Canon MR-14EX.

### Measurements

Six main characteristics of the new species were measured: body length (measured from anterior margin of head to end of cercus, thus excluding the antenna), index *t_2_* : *mt_2_* (= ratio of length of mid tibia : length of mid basitarsus), wing length (from wing base to wing tip), wing width (maximum width), *C-index* (*Cs_2_* : *Cs_3_*) (= ratio of length of 2nd costal sector : length of 3rd costal sector) and index *rm\dm-cu* : *dm-cu* (= ratio of length of section between *rm* and *dm-cu* on discal cell : length of *dm-cu*). All type specimens were measured.

### Presentation of faunistic data

Label data of primary-type specimens are presented strictly verbatim including information on form and colour of all associated labels. Data from paratypes of the new species and also from formerly unpublished non-type specimens are standardized and presented in full. For data of paratypes or paralectotypes of other species and formerly published records original publications are cited. Phenological and other biological information obtained from the material examined and literature are given in the Biology paragraph; data on distributions are presented as summarized by Roháček et al. (2001) and Marshall et al. (2011).

### Morphological terminology

**Figures 2–6.**
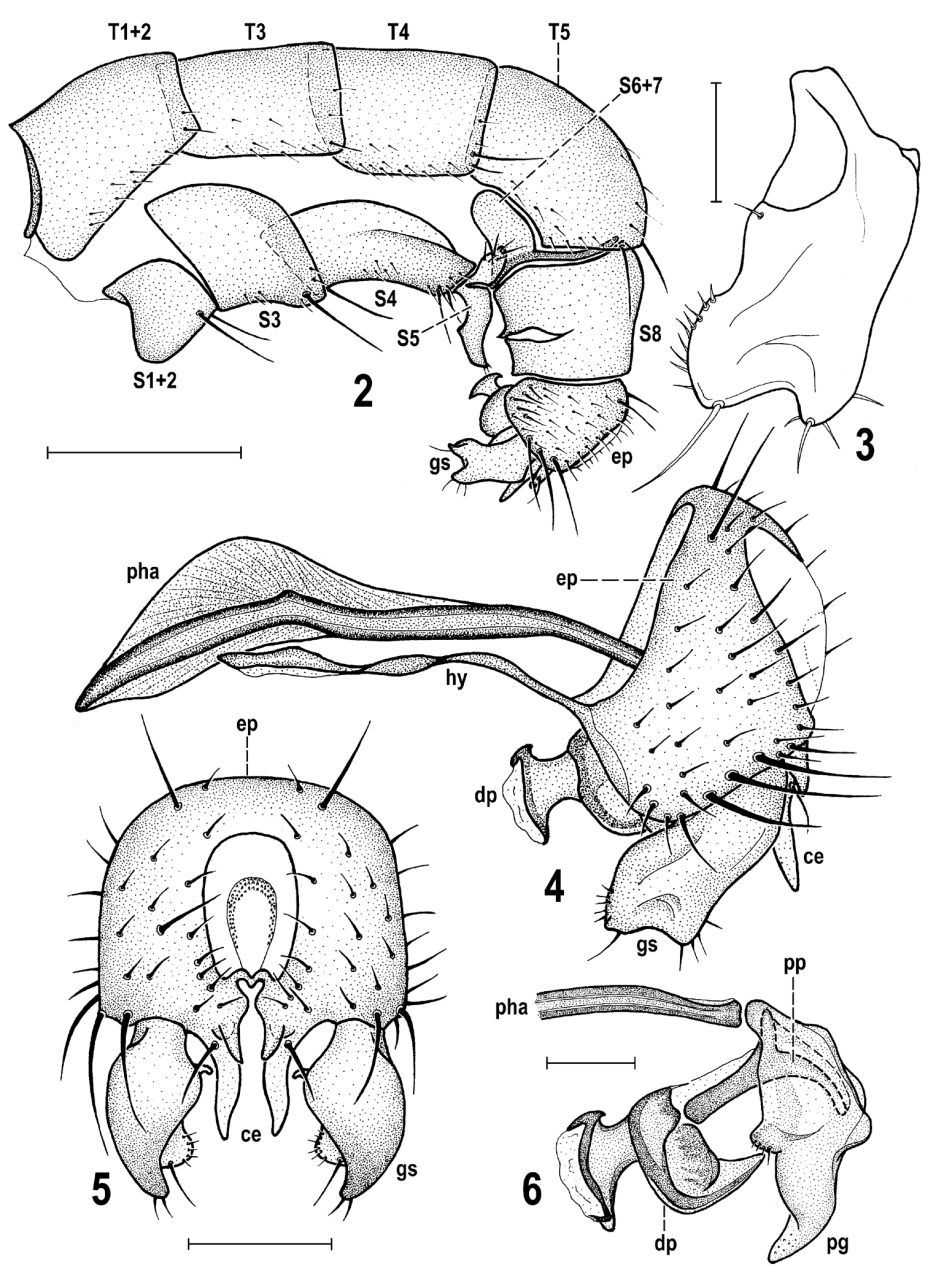
*Herniosina bequaerti* (Villeneuve), male (Czech Republic: Bohemia). **2** Abdomen, laterally **3** Gonostylus, laterally **4** Genitalia, laterally **5** External genitalia, caudally **6** Aedeagal complex (phallapodeme partly omitted), laterally. Scales: 0.5 mm (**2**), 0.1 mm (**3, 6**), 0.2 mm (**4, 5**). For abbreviations see pp. 73–74. Adapted from Roháček (1978).

**Figures 7–11.**
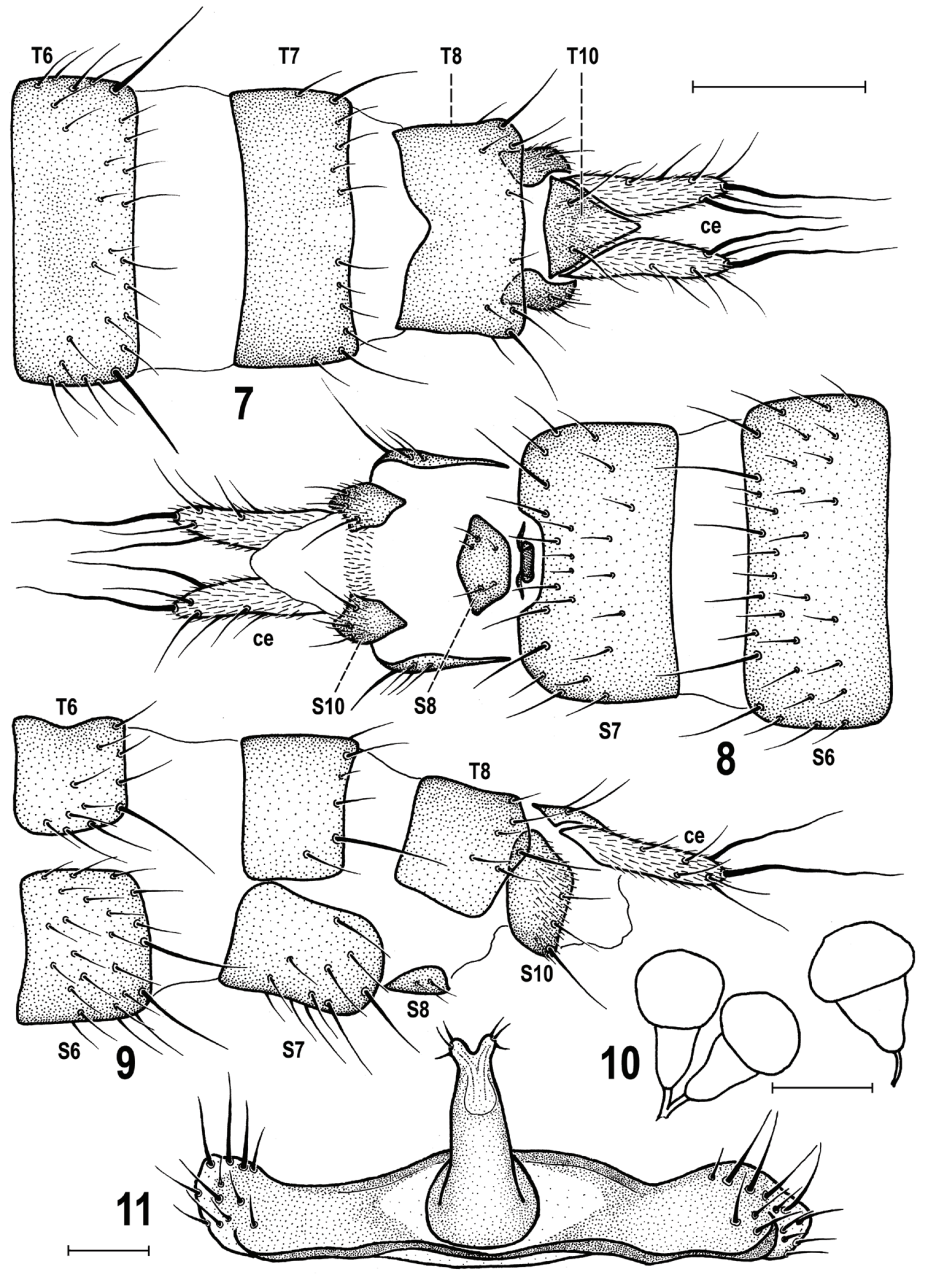
*Herniosina bequaerti* (Villeneuve), female, male (Czech Republic: Bohemia). **7** Female postabdomen, dorsally **8** Ditto, ventrally **9** Ditto, laterally **10** Spermathecae **11** Male *S5*, ventrally. Scales: 0.2 mm (**7–9**), 0.1 mm (**10, 11**). For abbreviations see pp. 73–74. Adapted from Roháček (1978, 1983).

Morphological terminology follows that used for Sphaeroceridae by Roháček (1998) in the Manual of Palaearctic Diptera including terms of the male hypopygium. The „hinge“ hypothesis of the origin of the eremoneuran hypopygium, re-discovered and documented by Zatwarnicki (1996), has been accepted and, therefore, the following synonymous terms of the male genitalia (emanating from other hypotheses) need to be listed (terms used first): ejacapodeme = ejaculatory apodeme, epandrium = periandrium, medandrium = intraperiandrial sclerite, phallapodeme = aedeagal apodeme. Morphological terms of the male postabdomen and genitalia are depicted in Figs 2, 4–6, those of the female postabdomen in Figs 7–9. Abbreviations of morphological terms used in text and illustrations are listed below.

### Abbreviations of morphological terms used in text and/or figures

*A_1_* anal vein

*ac* acrostichal (seta)

*ads* additional (setulae) on frons

*C* costa

*ce* cercus

*Cs_2_*, *Cs_3_* 2rd, 3th costal sector

*CuA_1_* cubitus

*dc* dorsocentral (seta)

*dm* discal medial cell

*dm-cu* discal medial-cubital (= posterior, tp) cross-vein

*dp* distiphallus

*ea* ejacapodeme

*ep* epandrium

*f_1_*, *f_2_*, *f_3_* fore, mid, hind femur

*g* genal (seta)

*gs* gonostylus

*hu* humeral (= postpronotal) (seta)

*hy* hypandrium

*ifr* interfrontal (seta)

*M* media

*mt_2_* mid basitarsus

*oc* ocellar (seta)

*occe* outer occipital (seta)

*occi* inner occipital (seta)

*ors* fronto-orbital (seta)

*pg* postgonite

*pha* phallapodeme

*pp* phallophore

*pvt* postvertical (seta)

*R_1_* 1st branch of radius

*R_2+3_* 2nd branch of radius

*R_4+5_* 3rd branch of radius

*r-m* radial-medial (= anterior, ta) cross-vein

*S1-S10* abdominal sterna

*sc* scutellar (seta)

*stpl* sternopleural (= katepisternal) (seta)

*T1-T10* abdominal terga

*t_1_*, *t_2_*, *t_3_* fore, mid, hind tibia

*va* ventroapical seta on t_2_

*vi* vibrissa

*vte* outer vertical (seta)

*vti* inner vertical (seta)

## Results

### Genus Herniosina

Taxon classificationAnimaliaDipteraSphaeroceridae

Roháček, 1983

Herniosina Roháček, 1983: 18 (feminine). – Roháček 1983: 18-21 [diagnosis, key, revision of European species, illustr]; Roháček 1993: 186 [taxonomy, key, illustr.]; Roháček 1998: 487 [diagnosis in key, illustr.]; Roháček et al. 2001: 148 [catalog].
Herniosina Roháček, 1982: 221 [nomen nudum, phylogeny]. Type species. Leptocera (Limosina) Bequaerti Villeneuve, 1917, original designation.

#### Diagnosis.

*pvt* absent; 3–5 *ifr*; 2–5 minute *ads* inside and below *ors*; *g* small; 2 *hu*, the internal reduced to microseta; 2 postsutural *dc*, the anterior short; *ac* setulae in 6–10 rows on suture, the medial prescutellar *ac* pair more or less enlarged; 2 *stpl*, the anterior very small, hair-like or absent; scutellum large, rounded triangular to trapezoidal; *t_2_* chaetotaxy as in Figs 16, 18, 57, 61, in male ventrally with a row of short spine-like setae and with reduced *va*, in female with anteroapical and *va* setae long (Fig. 18); male *f_2_* ventrally with a row of more or less thickened setae (Figs 18, 54) in basal half; *C* not extended beyond apex of *R_4+5_*; *R_4+5_* sinuate but apically almost straight; *dm* cell long and its posterior outer corner often rounded; alula relatively small and narrow; female postabdomen relatively (compared to preabdomen) narrow and telescopically retractile; male abdomen terminally strongly down-curved in consequence of enlarged *T5* and *S8* (Figs 1, 2, 15); male *S1+2* protruding in a slightly (Figs 47, 51) to strongly convex bulge (Figs 1, 15); male *S3* and *S4* with anterior corners lobe-shaped (= sclerites anteromedially more or less deeply emarginate, Figs 17, 41); male *S5* reduced, very transverse and band-shaped but posteromedially with a pair of projections (Figs 36, 65) which can be basally fused (Fig. 19) and/or prolonged (Fig. 11); epandrium with a row of robust long lateral setae at ventral margin and (usually) 1 longer dorsolateral seta (Figs 4, 28); male cerci modified to long, slender (usually doubled) processes (Figs 5, 21, 38, 50) below anal opening; gonostylus with a small (Fig. 5) to distinctive internal process (see Figs 22, 50); phallophore relatively long, anteriorly movably attached to dorsal side of distiphallus and projecting (epiphallus-like) posteroventrally (25, 26); distiphallus apically funnel-shaped and ventromedially projecting posteriorly in an unpaired process (Figs 26, 52); postgonite relatively robust, with small setulae only; ejacapodeme absent or strongly reduced (Fig. 53); female *T8* dorsomedially compact, paler pigmented or divided; female *T10* triangular, with a pair of long setae and some micropubescence; female *S8* reduced to a small sclerite with a few setulae (Figs 8, 33, 62); female *S10* short, strip-like, horseshoe- or V-shaped (Figs 33, 62, or medially divided into 2 sclerites (Fig. 8); female genital chamber membranous, lacking internal sclerites; spermathecae (2+1) pyriform (Fig. 10) to bulbous (Figs 59, 60); female cerci dark, long and slender, each with 2 long (apical and dorsopreapical) sinuous setae and a few shorter hairs.

**Figures 12–14.**
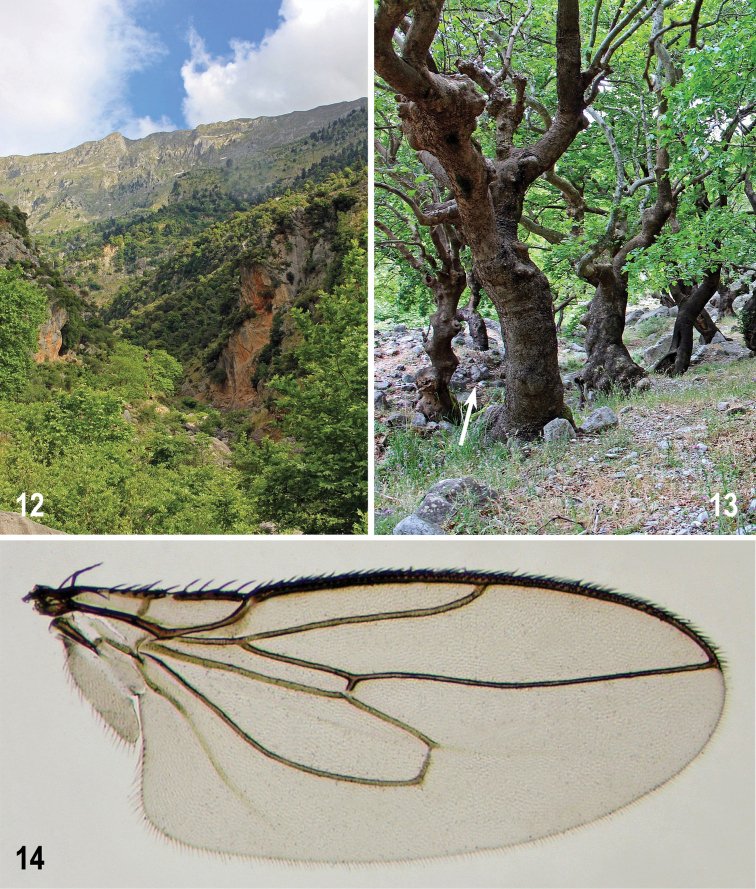
*Herniosina erymantha* sp. n., male holotype and its habitat (Greece: Peloponnese). **12** Valley above Alepochori in Erimanthos Mts, habitat in the type locality **13** Microhabitat (arrow) from where the holotype was sifted **14** Right wing (length 1.87 mm). Photographs by J. Roháček.

**Figures 15–19.**
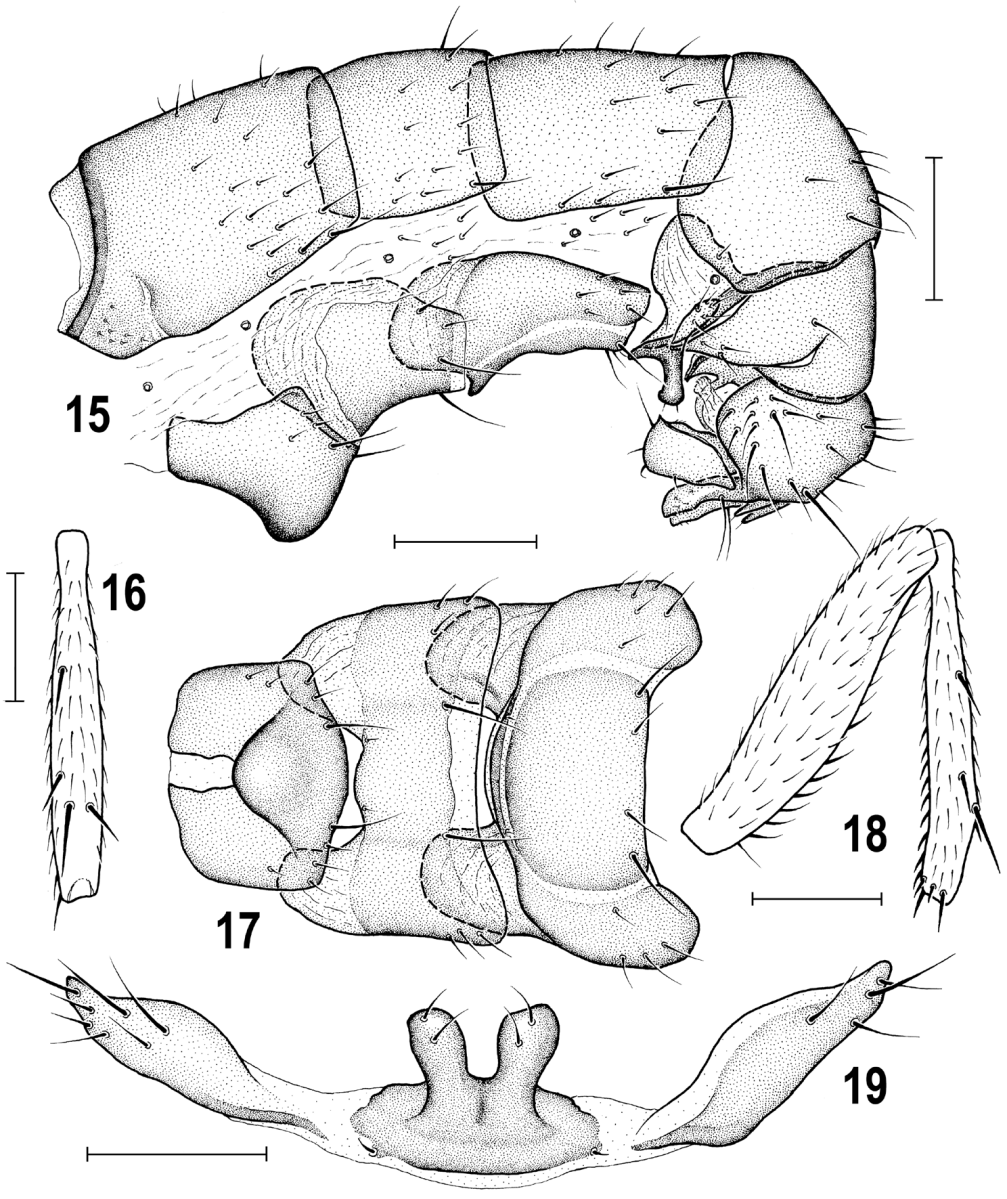
*Herniosina erymantha* sp. n., male holotype (Greece: Peloponnese). **15** Abdomen, laterally **16** Mid tibia, dorsally **17** Preabdominal sterna, ventrally **18** Mid femur and tibia, laterally **19** *S5*, ventrally. Scales: 0.2 mm (**15–18**), 0.1 mm (**19**).

**Figures 20–26.**
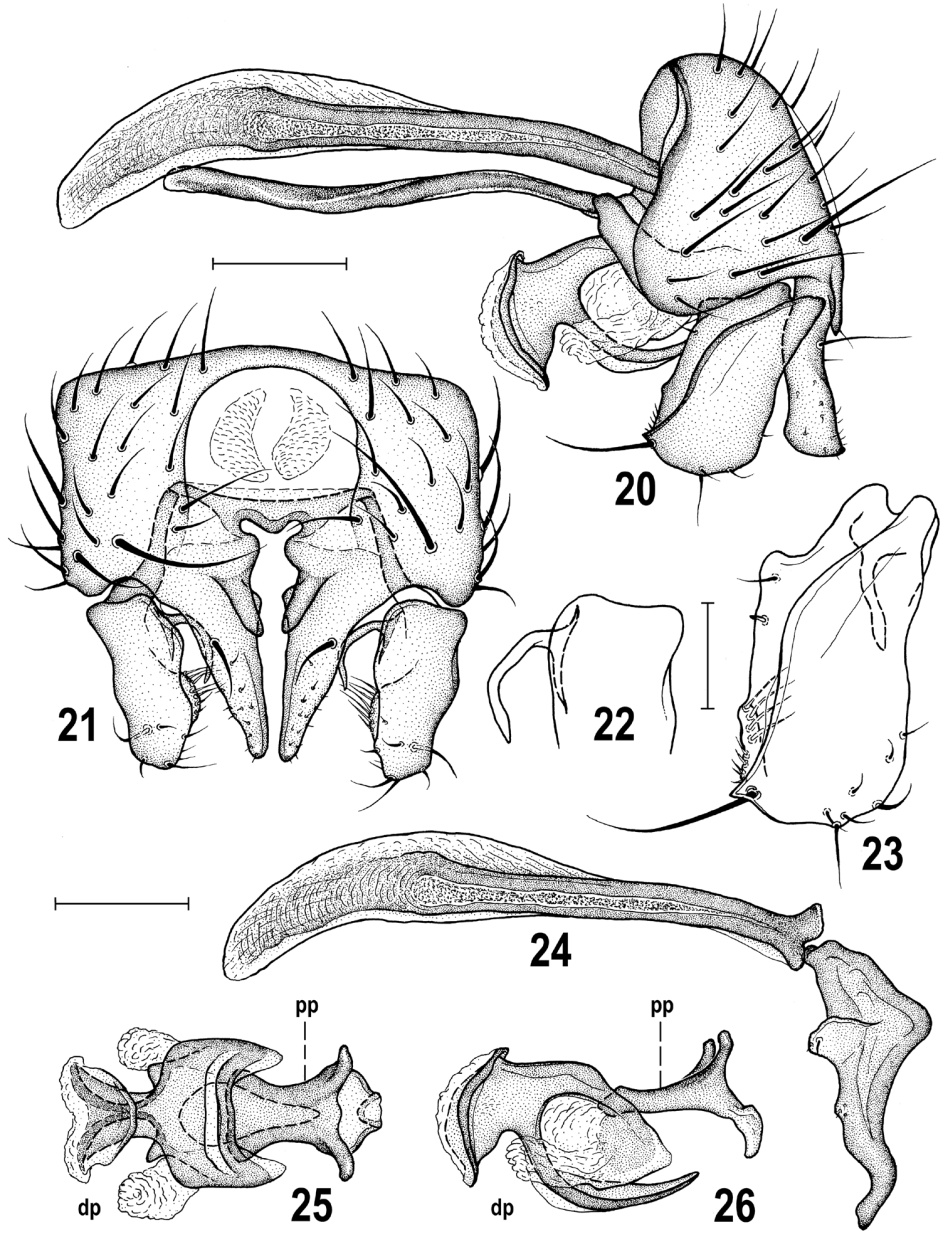
*Herniosina erymantha* sp. n., male holotype (Greece: Peloponnese). **20** Genitalia, laterally **21** External genitalia, caudally **22** Dorsal half of gonostylus, caudally **23** Gonostylus, laterally **24** Phallapodeme and postgonite, laterally **25** Aedeagus, dorsally **26** Ditto, laterally. Scales: 0.1 mm (**20, 21, 24–26**), 0.05 mm (**22, 23**). For abbreviations see pp. 73–74.

**Figures 27–30.**
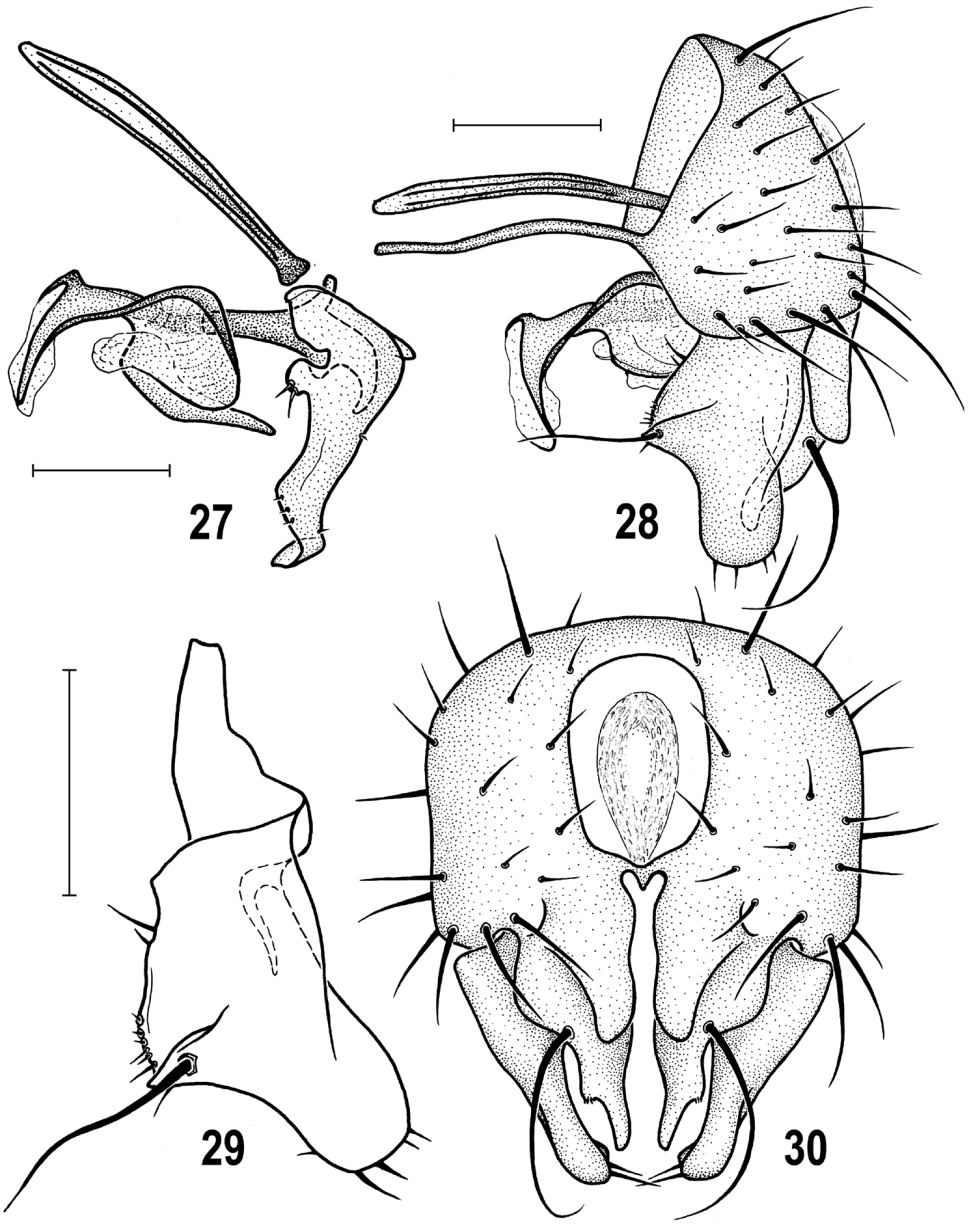
*Herniosina horrida* (Roháček), male paratype (Slovakia). **27** Aedeagal complex, laterally. **28** Genitalia, laterally **29** Gonostylus, laterally **30** External genitalia, caudally. All scales: 0.1 mm. Adapted from Roháček (1978).

**Figures 31–36.**
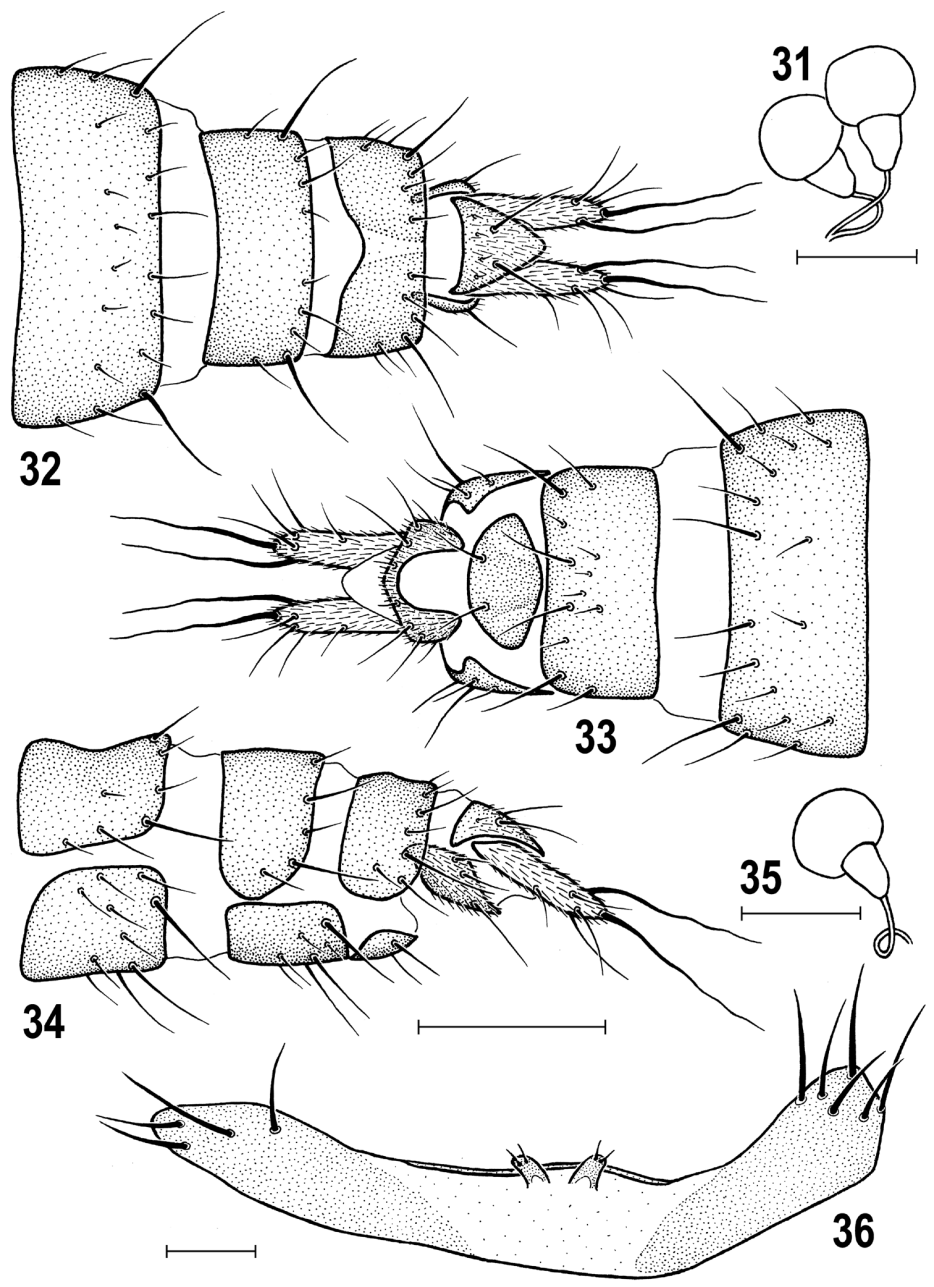
*Herniosina horrida* (Roháček), female and male paratypes (Slovakia). **31** Spermathecae **32** Female postabdomen, dorsally **33** Ditto, ventrally **34** Ditto, laterally **35** Spermatheca **36** Male *S5*, ventrally. Scales: 0.1 mm (**31, 35, 36**), 0.2 mm (**32–34**). Adapted from Roháček (1978).

**Figures 37–39.**
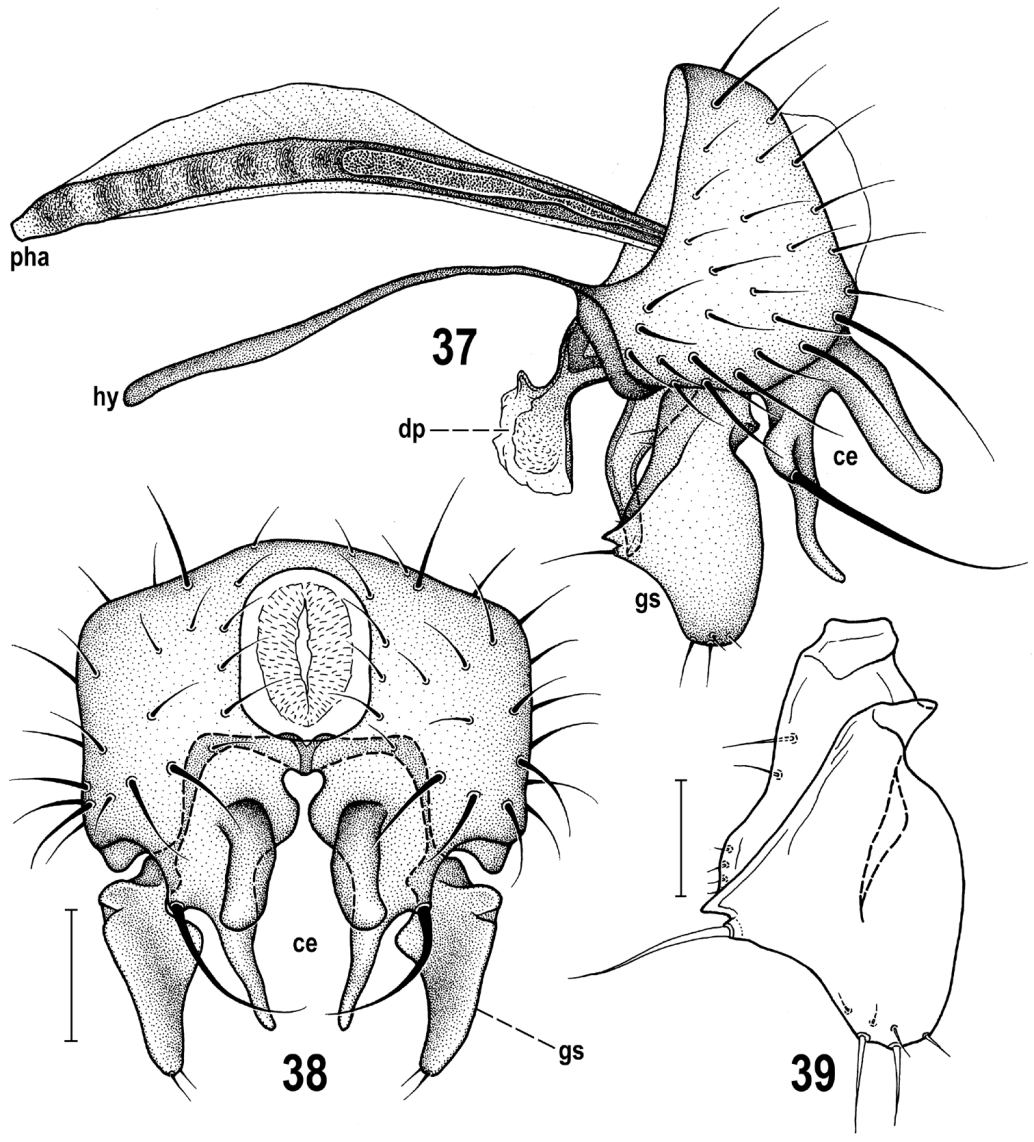
*Herniosina pollex* Roháček, male paratype (Slovakia). **37** Genitalia, laterally **38** External genitalia, caudally **39** Gonostylus, laterally. Scales: 0.1 mm (**37, 38**), 0.05 mm (**39**). For abbreviations see pp. 73–74. Adapted from Roháček (1993).

**Figures 40–42.**
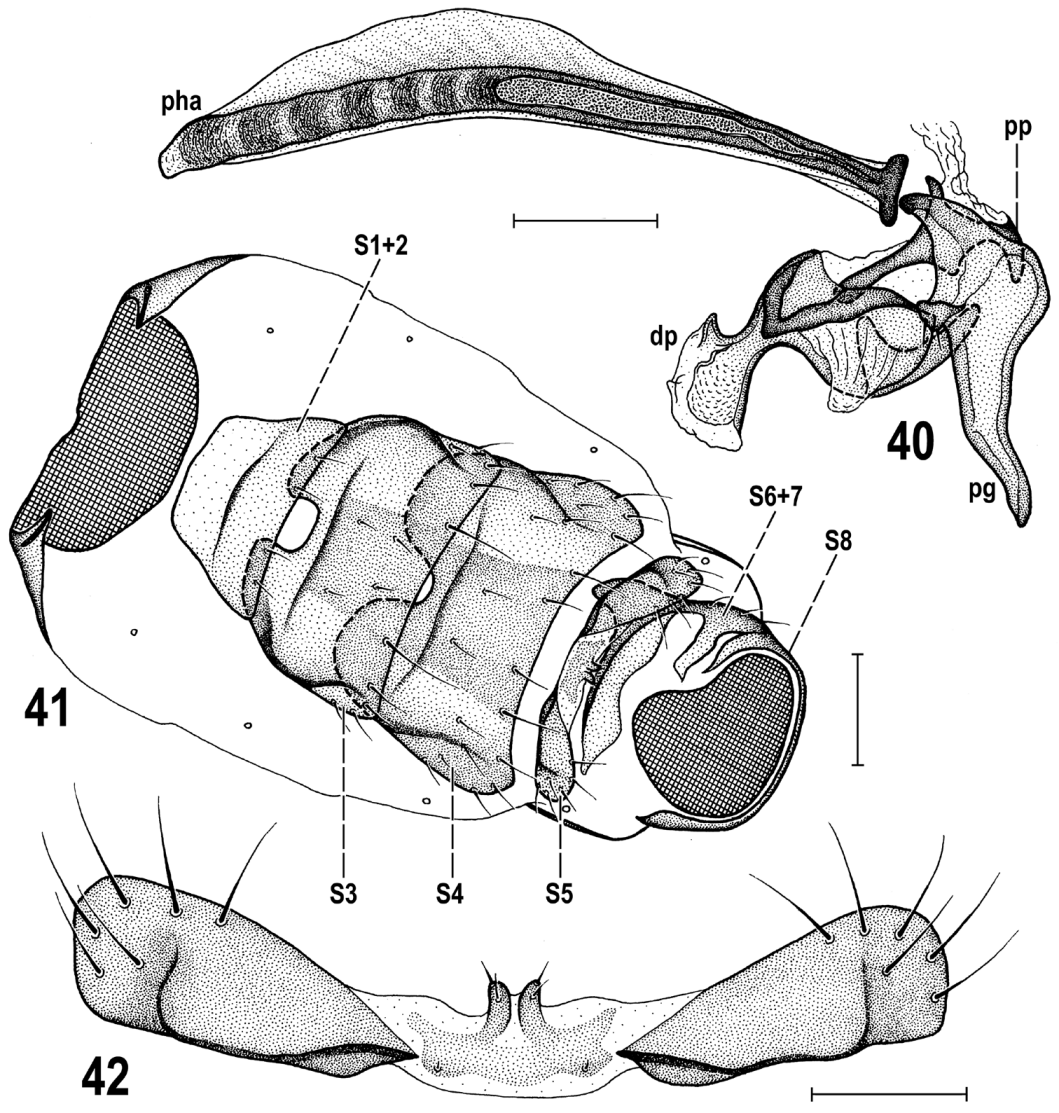
*Herniosina pollex* Roháček, male paratype (Slovakia). **40** Aedeagal complex, laterally **41** Abdomen, ventrally (genitalia removed) **42** Male *S5*, ventrally. Scales: 0.1 mm (**40, 42**), 0.2 mm (**41**). For abbreviations see pp. 73–74. Adapted from Roháček (1993).

**Figures 43–46.**
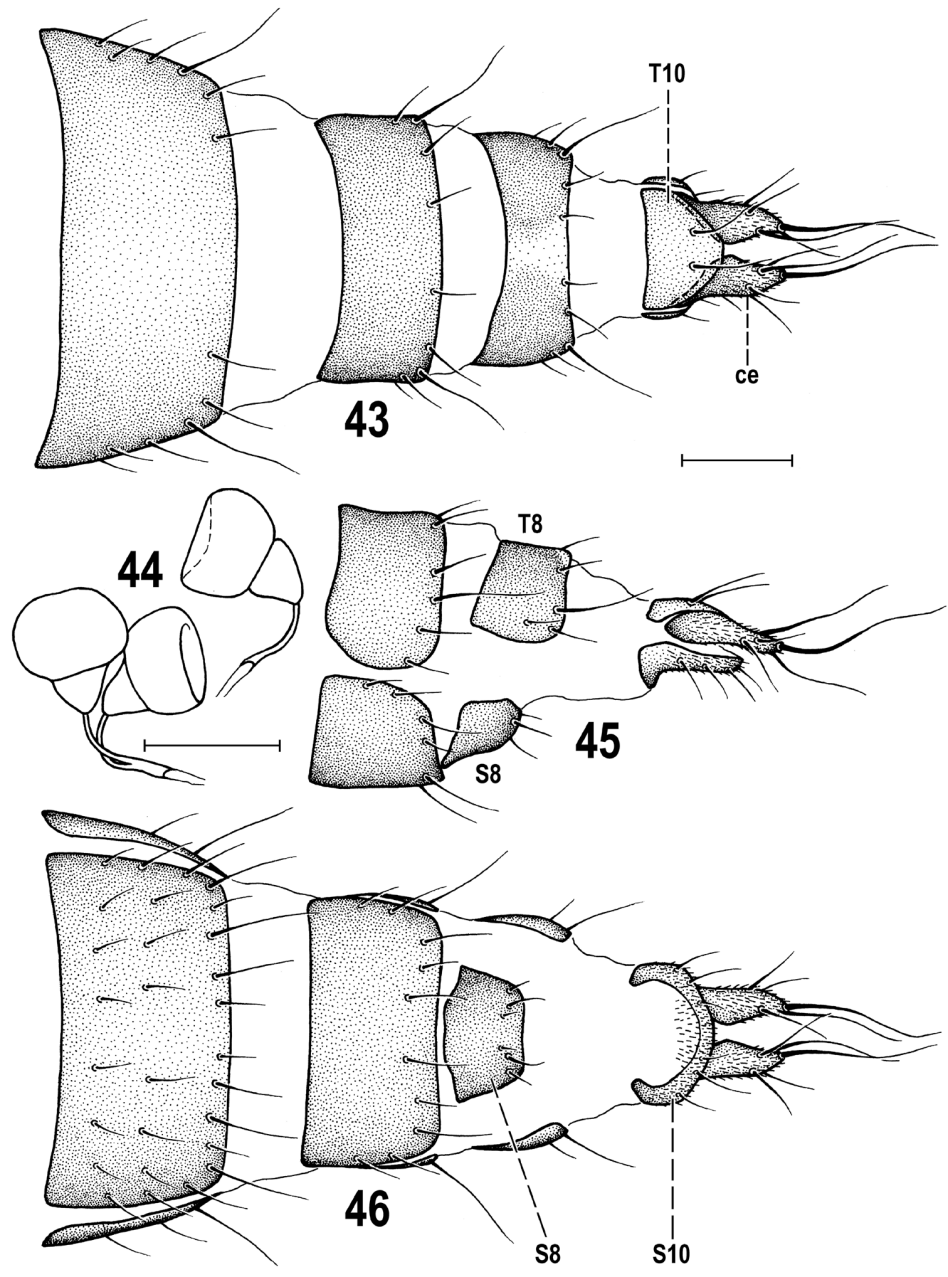
*Herniosina pollex* Roháček, female paratype (Slovakia). **43** Postabdomen, dorsally **44** Spermathecae **45** End of postabdomen, laterally **46** Postabdomen, ventrally. All scales: 0.1 mm. For abbreviations see pp. 73–74. Adapted from Roháček (1993).

**Figures 47–48.**
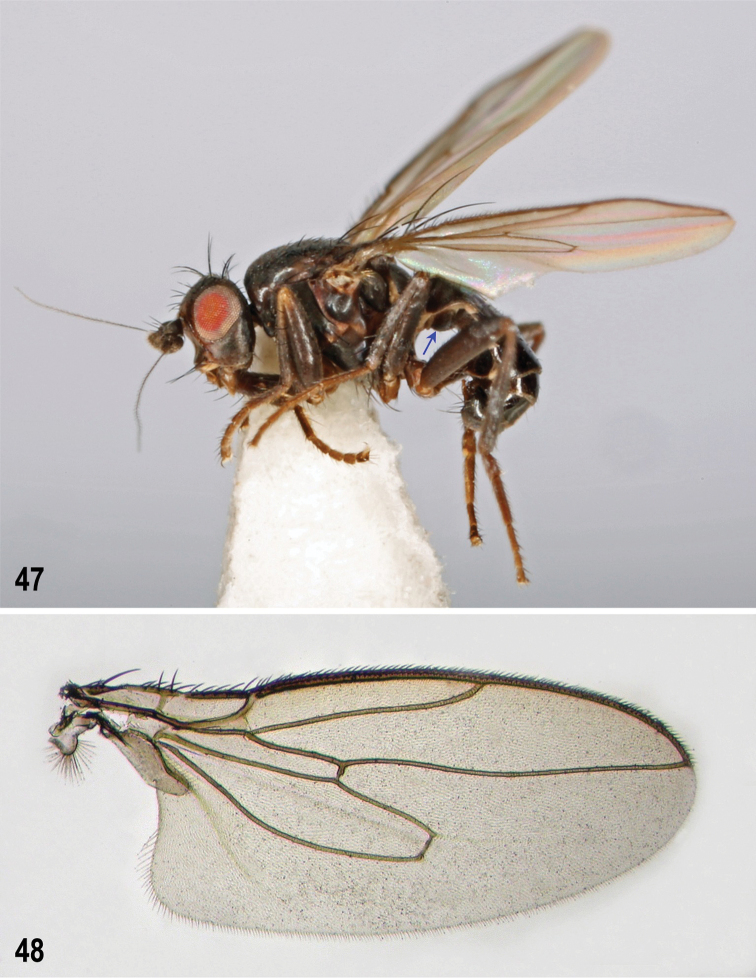
*Herniosina hamata* sp. n., male holotype (Cyprus). **47** Male, laterally, S1+2 arrowed **48** Right wing (length 2.24 mm). Photographs by J. Roháček.

**Figures 49–56.**
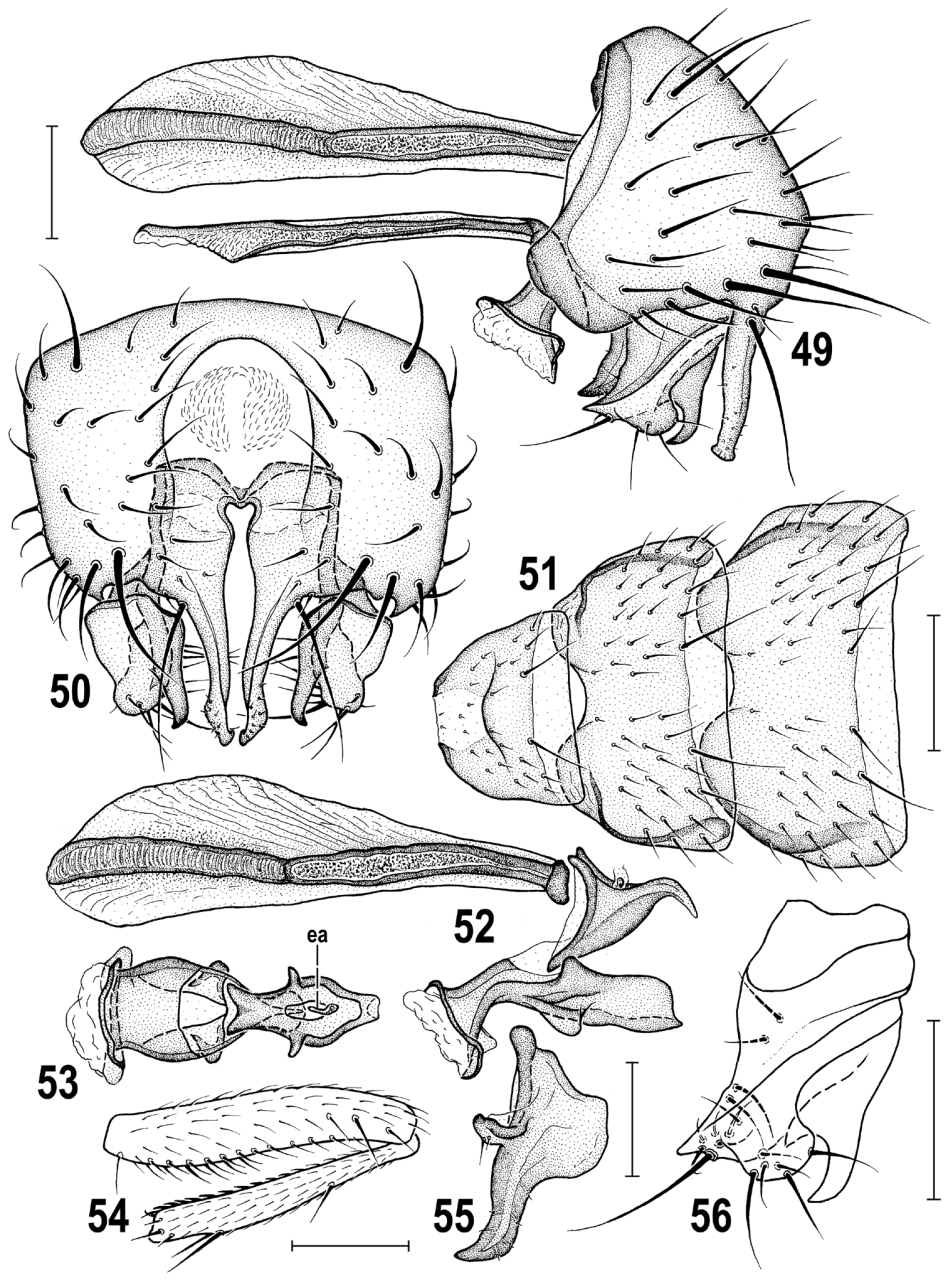
*Herniosina hamata* sp. n., male paratype (Cyprus). **49** Genitalia, laterally **50** External genitalia, caudally **51** Preabdominal sterna, ventrally **52** Phallapodeme and aedeagus, laterally **53** Aedeagus, dorsally **54** f_2_ and t_2_ anteriorly **55** postgonite, laterally **56** Gonostylus, laterally. Scales: 0.1 mm (**49, 50, 52, 53, 55, 56**), 0.2 mm (**51, 54**). For abbreviations see pp. 73–74.

**Figures 57–65.**
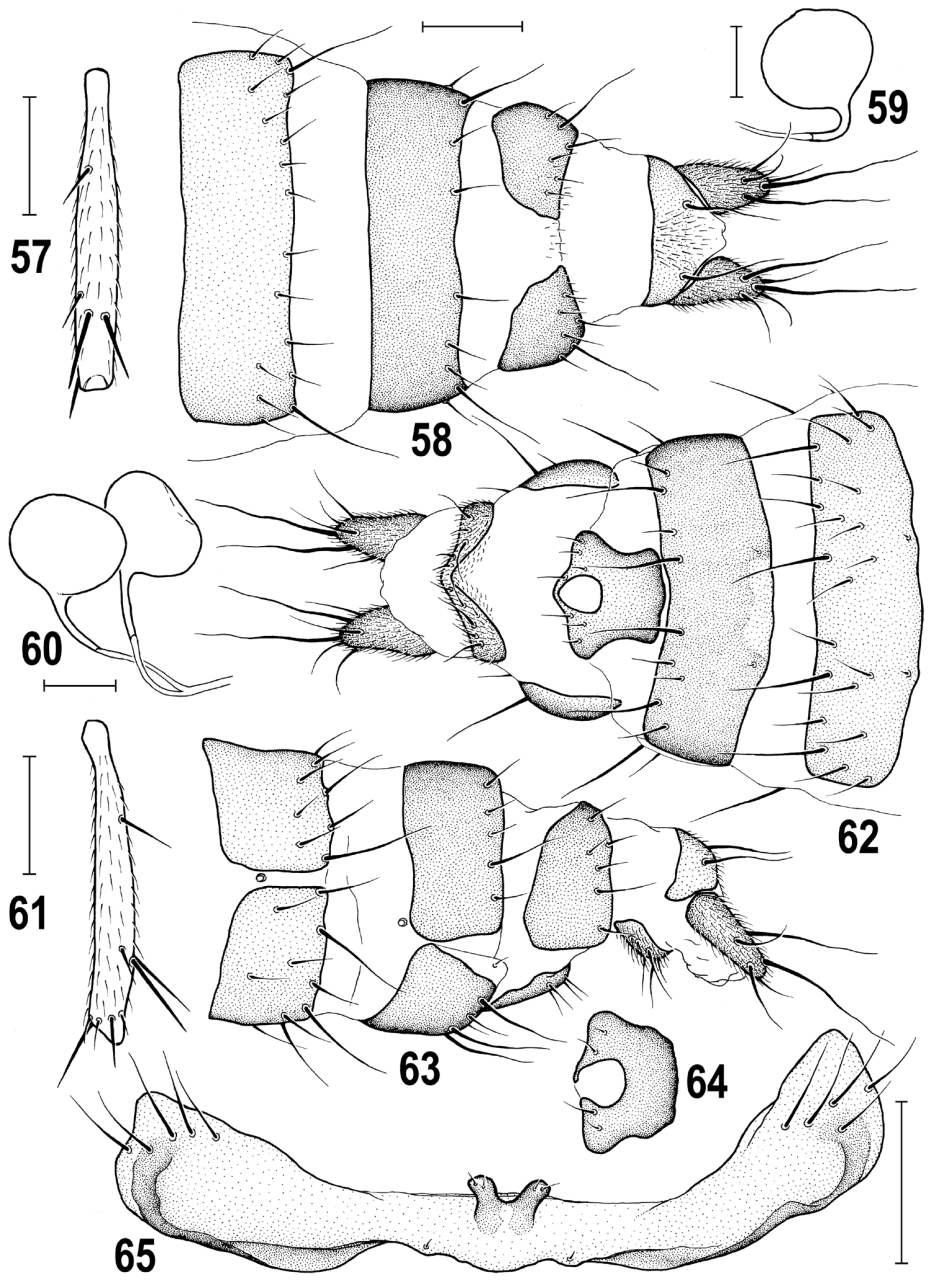
*Herniosina hamata* sp. n., female and male paratypes (Cyprus). **57** Female t_2_, dorsally **58** Female postabdomen, dorsally **59, 60** Spermathecae **61** Female t_2_, anteriorly **62** Female postabdomen, ventrally **63** ditto, laterally **64** female S8, ventrally **65** male S5, ventrally. Scales: 0.2 mm (**57, 61**), 0.1 mm (**58, 62–65**), 0.05 mm (**59, 60**).

#### Discussion.

The genus *Herniosina* can be identified by the key to European (Roháček 1983) and/or Palaearctic Limosininae (Roháček 1998). It seems to be best recognized by combination of apomorphic characters in the male abdomen and terminalia (postabdomen strongly down-curved, *S1+2* bulging, *S5* strongly reduced, cerci modified to peculiar projections, both distiphallus and phallophore projecting posteroventrally) and the plesiomorphic formation of the female postabdomen (relatively narrow and telescopic, no internal sclerites) having reduced *S8*. Although the diagnosis of the genus has to be somewhat modified (see above) with respect to the inclusion of the two new species described below, the genus remains to be a very compact monophyletic group if only Palaearctic species (reviewed here) are included.

However, Marshall (1987) described a very peculiar Limosinine species from USA (New Hampshire) and placed it as a tentative member of *Herniosina* on the basis of its bulging male *S1+2* although this *Herniosina voluminosa* Marshall, 1987 differs very markedly from all other *Herniosina* species in many characters, lacking all other synapomorphies of the genus as originally delimited, including the modified male preabdominal sterna, reduced male *S5*, enlarged *T5*, projecting male cerci, form of phallophore, general shape of spermathecae etc. I have examined male and female paratypes of *Herniosina voluminosa* kindly donated by S. A. Marshall to SMOC (USA: New Hampshire: Coos Co., 3 mi. NE East Inlet Dam, Norton Pool, flight interception trap, 12.-24.vi.1986, 1♂, 25.vi.-9.vii.1986, 1♀, D. S. Chandler leg.) and found that the male *S1+2* of *Herniosina voluminosa* is differently (posteromedially) protruding and somewhat bilobed (see also Marshall 1987: Fig. 1) suggesting that this modification of *S1+2* evolved independently and hence cannot be a synapomorphy of this Nearctic species and *Herniosina* s. str. species. Also its extremely enlarged aedeagal complex (with enormous distiphallus, phallophore and postgonite being several times larger than epandrium) and quite differently formed female terminalia (with *S8* large and transverse, internal spectacles-shaped sclerite developed, spermathecae elongate and transversely wrinkled and *T10* fused with base of cerci) clearly demonstrate that these taxa cannot be congeneric. Inasmuch as it seems that both known *Apteromyia* species (cf. Marshall and Roháček 1982) are apparently more closely allied to the Palaearctic species of *Herniosina* than is *Herniosina voluminosa* (see below) the latter species is excluded from *Herniosina* here to render the genus monophyletic. It is therefore suggested to establish a new genus for the removed *Herniosina voluminosa* in the near future.

When describing the genus, Roháček (1982: 221, 1983: 18) placed *Herniosina* in the *Limosina* genera-group and discussed its affinity as being either a sister-group of the genus *Apteromyia* Vimmer, 1929 or sister-group of a clade comprising besides the latter genus also two other members of the *Limosina* genera-group, viz. *Limosina* Macquart, 1835 and *Gigalimosina* Roháček, 1983. Subsequently, Marshall & Roháček (1982) considered the former hypothesis to be more probable pointing out similarly modified (projecting) male cercus and setosity of epandrium in the Nearctic species *Apteromyia newtoni* Marshall & Roháček, 1982 and *Herniosina* species. Based on the present study the relationships of *Herniosina* and *Apteromyia* is supported by four supposedly synapomorphic characters (all in the male genitalia): epandrium with a series of robust ventral lateral setae; male cerci modified to compact processes below anal fissure, distiphallus with unpaired ventromedial lobe projecting posteriorly; phallophore anteriorly slender and elongately projecting, movably attached to dorsal side of distiphallus.

#### Species included.

*Herniosina bequaerti* (Villeneuve, 1917), *Herniosina erymantha* sp. n. (described here), *Herniosina horrida* (Roháček, 1978), *Herniosina pollex* Roháček, 1993 and *Herniosina hamata* sp. n. (described here). Hitherto, *Herniosina* species are only known from the W. Palaearctic area, including that recorded as Herniosina sp. cf. horrida from Israel by Papp and Roháček (1988). The latter record, based on two females only, may belong to *Herniosina horrida* or, more probably, to an additional unnamed species but its description is pending further study of (hitherto unknown) males.

### Herniosina bequaerti

Taxon classificationAnimaliaDipteraSphaeroceridae

(Villeneuve, 1917)

Figs 1 , 2–6 , 7–11

Leptocera (Limosina) Bequaerti Villeneuve, 1917: 143 [both sexes]. Type locality: The Netherlands, Maestricht, St. Pietersberg.
Leptocera (Scotophilella) Bequaerti. – Duda 1925: 154 [subgeneric combination].
Limosina (Limosina) Bequaerti. – Duda 1938: 110 [generic combination, illustr.].
Leptocera bequaerti . – Goddard 1938: 240–241 [puparium, illustr.].
Limosina bequaerti . – Roháček 1978: 55 [redescription, genitalia, illustr.]; Papp 1984: 96 [Palaearctic catalog].
Herniosina bequaerti . – Roháček 1982: 260–263 [illustr.]; Roháček 1983: 19 [generic combination, redescription, phylogenetic notes]; Roháček 1993: 191 [key]; Skidmore 1993: 8, 16 [puparium, illustr.]; Roháček et al. 2001: 148 [catalog]; Marshall et al. 2011: 243 [catalog].
Limosina (Scotophilella) herniata Duda, 1918: 108 [both sexes, illustr.]. Type locality: Austria, “Styriae Alpes”. – Duda 1924: 194 [synonymy].

#### Type material.

*Leptocera (Limosina) Bequaerti* Villeneuve: Lectotype ♂ (designated by Roháček 2001: 471), labelled: „Maestricht, S^t^Pietersberg, 5-IX-12“ (obverse), „grot“ (reverse), „Limosina sp. III, bu L. nana Rdi.“ (handritten by ?), „Limosina Bequaerti Villen.“ (Villeneuve’s handwriting) and „Leptocera (Limosina) Bequaerti Villen., J. Roháček des. 2000, ♂ Lectotypus“ (red label). Paralectotype ♀ labelled as lectotype but lacking the label „Limosina sp. III ...“. Lectotype with genit. prep., paralectotype intact, both deposited in MNHN.

Limosina (Scotophilella) herniata Duda: Lectotype ♂ (designated by Roháček, 1983: 19), labelled: „Styriae Alpes Strobl“, „L. rufilabris Stenh. ♂ 23/9“, „52 138“, „nova spec. Herniata mihi det Duda“ (pink label), deposited in ZMHB. Paralectotypes: 2♀, labelled: „No. 200 Wypustek“ and „herniata ♀ det Duda“, deposited in MMBC).

#### Other material examined.

90♂78♀ – BELGIUM: 5♂5♀ (ISNB), for localities see Roháček (1978). CZECH REPUBLIC: 80♂67♀ (JRO, MMBC, NMPC, SMOC), for localities see Roháček (1978, 1980, 1983, 1984); additional data: SE Bohemia: Palupín nr. Strmilov (distr. Jindřichův Hradec), in cellar, 19.viii.1991, 55♂ 39♀, J. Roháček leg. (JRO); N Moravia: Vidnava env. (distr. Šumperk), nest of *Talpa europaea*, 28.iii.1985, 1♀, J. Roháček leg. (SMOC). FINLAND: 1♀ (MZHF), for locality see Roháček (1983). ITALY: 1♂ (MSNV), for locality see Roháček (1983). SLOVAKIA: 4♂5♀ (SMOC), for localities see Roháček (1983, 1986, 1994, 2009); addtional data: NE Slovakia: Regetovka env. (distr. Bardejov), sifting decayed grass in runs of *Microtus agrestis*, 10.x.1985, 1♂, J. Roháček leg. (SMOC).

#### Diagnosis.

Largest Palaearctic species on the average (body length: male 2.26–2.85 mm, female 2.22–3.05 mm), with lightest head (ochreous to reddish brown anteriorly). Male: abdomen with large and long *T5* and *S8* (Fig. 2); *S1+2* strongly bulging (Figs 1, 2); *S5* with longest (in lateral view sinuate) medial, apically forked, process (Figs 2, 11); epandrium with a long dorsolateral seta (Fig. 4); cerci relatively shortly but acutely double-projecting (Fig. 5); gonostylus rather simple (Fig. 3), ventrally somewhat emarginate, with small and short internal subdorsal projection (cf. Fig. 5); hypandrial rod long and slender (Fig. 4); phallapodeme long, with large dorsal keel (Fig. 4); postgonite short and robust; distiphallus with short lateral and ventral lobes and robust funnel-shaped apex (Fig. 6). Female: postabdomen slender, with relatively narrow sclerites of 6th–8th segment (Figs 7, 8); *T8* complete and entirely pigmented (Fig. 7); *S8* small, subtrapezoidal, with a small anterior structure (Fig. 8); spermathecae pyriform with conical base (Fig. 10); *S10* medially divided (Fig. 8); cerci long and slender (Figs 7–9).

#### Biology.

*Herniosina bequaerti* is closely associated with subterranean habitats, such as caves (Czižek 1916; Duda 1918, 1938; Papp & Plachter 1976), cellars (Pax & Maschke 1935; Roháček 1978) and burrows, runs and nests of small mammals, including those of rabbits (*Oryctolagus cuniculus*), rats (*Rattus norvegicus*), hedgehogs (*Erinaceus europaeus*), moles (*Talpa europaea*), shrews (*Sorex araneus*), mice (*Mus musculus*) and voles (*Microtus agrestis*, *Microtus* sp., *Arvicola terrestris*) (Duda 1918; Richards 1930; Goddard 1938; Roháček 1978, 1983; Rotheray 1991). Adults can be also caught by means of soil traps (Roháček 1980); only very scarcely (migrated specimens) they can be collected outside subterranean habitats (e.g. Grundmann 1991).

Because of being adapted to cold and the complete darkness in aphotic parts of caves and having the ability to develop under these conditions, Papp and Plachter (1976) classified *Herniosina bequaerti* as a troglophilous species. They found larvae consuming various decaying media, viz. dead animals, rotten vegetation including wood and/or mycelia of fungi and determined the length of its life-history (from egg to imago) within caves as 70–90 days and the life as an adult being 38 days on the average. The sometimes abundant occurrence of the species in cellars (see Roháček 1978 and material examined), caused by convenient conditions and a rich supply of larval food, can be considered a special case of synanthropy (Roháček 1983). In burrows of mammals the larvae develop in their droppings and other nest debris (Rotheray 1991) but obviously much more rapidly due to higher temperature. Adults can occur throughout the whole year. Puparia of *Herniosina bequaerti* were described by Goddard (1938) and Skidmore (1993) based on specimens found in mouse-runs and on subfossil specimens excavated in an archaeological site in Iceland respectively.

#### Distribution.

Widespread in Europe (Austria, Belgium, Czech Republic, Finland, Germany, Great Britain, Hungary, Iceland, Ireland, Italy, Latvia, Netherlands, Poland, Slovakia, Spain, Sweden, Switzerland) but surprisingly hitherto unrecorded from its SE part (Balkan peninisula).

### Herniosina erymantha sp. n.

Taxon classificationAnimaliaDipteraSphaeroceridae

http://zoobank.org/174E0AE5-52C3-417A-9AAF-3120652AAC8B

Figs 14 , 15–19 , 20–26

#### Type material.

Holotype ♂ labelled: „GREECE: NW Peloponnese: Alepochori 0.5 km SE 37°58'57"N, 21°48'10"E“, „590 m, 27.5.2015, sifting leaves under *Platanus*, J. Roháček leg.“, „Holotypus ♂ Herniosina erymantha sp. n., J. Roháček det. 2016“ (red label). The specimen is dry-mounted on pinned triangular card, with left wing and abdomen detached, genitalia dissected and all removed parts preserved in glycerine in coalesced plastic tube pinned below the specimen (SMOC).

#### Etymology.

The name of the new species is an adjective derived from the Erimanthos (= Lat. Erymanthos) Mts inasmuch as its type locality is situated in the western part of this montane range.

#### Description.

**Male.** Total body length 1.79 mm; general colour blackish brown with relatively sparse dark greyish brown microtomentum. Head blackish brown to brown. Frons largely blackish brown, brownish only at anterior margin, rather sparsely microtomentose. Occiput blackish brown and dark greyish brown microtomentose. Orbits, interfrontalia (poorly delimited) and ocellar triangle also greyish brown (not densely) microtomentose and duller than rest of frons; frontal triangle comparatively wide and shining. Cephalic chaetotaxy: *pvt* absent, only minute adpressed postocellar setulae behind ocellar triangle; *occe* and *occi* subequal and less than half length of *vte*; *vti* longest among frontal setae, *vte* and *oc* slightly shorter than *vti*; 2 strongly exclinate *ors*, both distinctly shorter than *oc*; only 3 relatively short *ifr*, the middle pair longest; 3–4 very minute *ads* inside and below *ors*; *g* weak, not longer than anterior peristomal setula; *vi* about as long as *vti*. Frontal lunule short, wide, similarly brown as anterior margin of frons. Face with cavities below antennae dark brown, rather shining; medial carina slightly elevated but distinct. Gena high, reddish only anteriorly, otherwise blackish brown, sparsely greyish microtomentose. Eye relatively small; its longest diameter about 2.2 times as long as smallest genal height. Antenna blackish brown, relatively long; its 3rd segment distinctly tapered apically both in dorsal and lateral view. Arista long, about 3.8 times as long as antenna, relatively long ciliate.

*Thorax* dark brown to blackish, mesonotum subshining due sparser microtomentum, pleuron lighter and dull. Suturae between pleural sclerites paler brown. Scutellum relatively large and long, rounded triangular. Thoracic chaetotaxy: 2 *hu* but internal reduced to microseta; 2 postsutural *dc*, anterior short and weak (only twice longer than ac microsetae), posterior strong but slightly shorter than basal *sc*; 8–10 rows of *ac* microsetae on suture; medial prescutellar *ac* pair somewhat prolonged; 2 strong *sc*, basal slightly longer than scutellum, apical about 1.6 times as long as basal; 2 *stpl* but anterior reduced to minute hair-like setula.

*Legs* dark brown, coxae, trochanters, knees and tarsi pale brown to ochreous. *f_1_* with relatively sparse setae in posterodorsal and posteroventral rows. *f_2_* with a row of curved but relatively short ventral setae in basal half (Fig. 18); *t_2_* ventrally with a long row of small dense spines and reduced *va* seta (shorter than anteroapical seta), see Fig. 18; dorsal chaetotaxy of *t_2_* as in congeners but posterodorsal seta in apical fourth somewhat shorter (Fig. 16). *t_2_* : *mt_2_* = 1.84.

*Wing* (Fig. 14) with pale brownish membrane and ochreous to dark brown veins. *C* hardly produced beyond apex of *R_4+5_*. *R_2+3_* slightly sinuate but apically distinctly upcurved to *C*; *R_4+5_* sinuate and its apical half almost straight. Discal cell (*dm*) relatively short and distally less tapered than in most relatives, with small process of *M* beyond *dm-cu*; posterior outer corner of dm not rounded but obtuse-angled. *A_1_* sinuate; anal lobe well developed; alula narrow but not acute. Wing measurements: length 1.87 mm, width 0.77 mm, *C-index* = 0.95, *rm\dm-cu* : *dm-cu* = 2.62. Haltere with ochreous stem and dark brown knob.

*Abdomen* blackish brown, with some sclerites brown. Preabdominal terga large, shining blackish brown, with only scarce greyish microtomentum, sparsely and shortly setulose. *T4* longer than *T3*; *T5* enlarged but less than that of *Herniosina bequaerti*, postabdomen strongly down-curved (Fig. 15). Preabdominal sterna as in Figs 15, 17: *S1+2* strongly bulging (Fig. 15) and anteromedially narrowly desclerotized (Fig. 17); *S3* and *S4* deeply anteriorly emarginate due to large lateral lobes (Fig. 17), with distinctive pigmentation; *S1+2*, *S3* and *S4* with sparse setae at posterior margins, with only medial (in *S4* submedial) pair longer. *S5* (Fig. 19) reduced and transversely strip-shaped, with setose lateral parts as in relatives but with distinctive dark medial part provided with a flattened (in lateral view knob-like, Fig. 15) and distally forked process having 2 setulae on each lobe of its digitiform terminal part (Fig. 19). *S6* and *S7* fused to form a complex sclerite on left side of postabdomen, narrow ventrally and dorsally but dilated laterally (Fig. 15) as in congeners. *S8* shorter and more tapered, with a slit left laterally and a few setae.

*Genitalia.* Epandrium (Figs 20, 21) of medium length but comparatively wide and more angular dorsolaterally (see Fig. 21) than those of relatives, and with a group of longer and stronger setae laterally and lateroventrally (posterior seta longest and most robust) but dorsolaterally without longer seta. Anal fissure slightly wider than high (Fig. 21), subcircular. Cerci fused with epandrium, posteroventrally projecting in 2 processes, one (more anterior) robust, long (about as long as gonostylus) and distally somewhat thickened and bearing 1 long seta in addition to a number of setulae, the other (more medial) small, elongately conical and simple (Figs 20, 21). Medandrium small (low), reduced but connected by long internal arms with gonostyli (Fig. 21), posteromedially fused with cerci. Hypandrium with very long and slender anteromedial rod-like apodeme (Fig. 20). Gonostylus (Figs 20–23) most resembling that of *Herniosina bequaerti* but ventrally simple (not emarginate in lateral view), with longer anteroventral seta, and with long, very slender, curved and apically lancet-shape dorsal internal process (cf. Fig. 22). Aedeagal complex (Figs 24–26) with long phallapodeme (as in *Herniosina bequaerti*) but having distinctly lower dorsal keel. Aedeagus also somewhat similar to that of *Herniosina bequaerti* but with funnel-shaped apex of distiphallus more robust, its lateral lobes and unpaired ventral process markedly longer and its postgonite more slender, with curved, slender and terminally blunt apex. Phallophore closely resembling that that of *Herniosina bequaerti*, anteriorly rod-like but dorsoventrally flattened, posteriorly projecting ventrally and hence epiphallus-like. Ejaculatory apodeme not observed.

**Female** unknown.

#### Discussion.

*Herniosina erymantha* sp. n. is only known from the male holotype and, consequently, for the evaluation of its relationships the female characters cannot be used. However, based on the male terminalia the species is a distinctive member of *Herniosina* unmistakeably recognizable from any other known congener. It differs from all relatives by the flattened, distinctively forked medial process of *S5* (Fig. 19), broad epandrium with strikingly rectangular dorsolateral corners without dorsolateral long seta and with broad (wider than high) anal fissure (Fig. 21), relatively simple sub-oblong gonostylus (Fig. 23) with very slender and long internal process (Fig. 22), distinctive cercus with long and robust, distally somewhat dilated but laterally flattened lateral process combined with short medial ones (Figs 20, 21) and characteristic postgonite (Fig. 24).

Judging from the construction of the male abdomen and male genitalia, *Herniosina erymantha* seems to be related to *Herniosina bequaerti* and *Herniosina horrida* sharing with them the following synapomorphies: strongly bulging *S1+2*; very slender and dorsally situated internal process of gonostylus. Its closest relative obviously is *Herniosina bequaerti* having similarly (albeit much more) prolonged and basally fused medial processes of *S5*, a small medial apically pointed process of cercus and more robust funnel-shaped apex of distiphallus.

#### Biology.

The holotype of *Herniosina erymantha* sp. n. was sifted towards the end of May from dead leaves under *Platanus* trees (see Fig. 13, arrow) in a valley of a montane brook in the western ridge of the Erimanthos Mts (Fig. 12). The microhabitat (layers of decaying leaves of broad-leaved trees) is similar to that known for *Herniosina horrida* and *Herniosina pollex* in Central Europe (see under these species).

#### Distribution.

Hitherto only known from Greece: NW Peloponnese.

### Herniosina horrida

Taxon classificationAnimaliaDipteraSphaeroceridae

(Roháček, 1978)

Figs 27–30

Limosina horrida Roháček, 1978: 51 [both sexes, illustr.]. Type locality: Slovakia, Veľká Fatra Mts., Suchá dolina (valley). – Papp 1984: 99 [Palaearctic catalog].
Herniosina horrida. – Roháček 1982: 265-266 [illustr.]; 1983: 20 [generic combination; redescription, phylogenetic notes]; 1993: 191 [key]; Roháček et al. 2001: 149 [catalog].

#### Type material.

Holotype ♂ labelled: „Slovakia centr. 27.6.1975, V. Fatra, Suchá dolina, J. Roháček leg., decayed hay“ (handwritten) and „Limosina horrida sp.n., J. Roháček det., holotypus ♂“ (framed handwriting), deposited in JRO ((intact, in ethanol). Allotype ♀ (JRO) and 7♂11♀ paratypes (all in ethanol, some with genit. prep.), with the same data, deposited in JRO and HNHM. For data of other paratypes (7♂18♀, JRO, SMOC) from the Czech Republic and Slovakia see Roháček (1978, 1983).

#### Other material examined.

18♂32♀ – AUSTRIA: 1♂2♀ (NHMW), for localities see Roháček (1993). CZECH REPUBLIC: 2♂17♀ (JRO, MBP, SMOC, UEBC), for localities see Roháček (1980, 1983, 1984, 1993, 1996); additional data: N Moravia: Vidnava env. (distr. Šumperk), sweeping undergrowth of deciduous forest, 10.vii.1984, 1♀; Hrubý Jeseník Mts, Velká kotlina valley, on excrement of red deer, 20.vi.1990, 1♂; Dlouhá Stráň env. (distr. Bruntál), mouth of muskrat (*Ondatra*) burrow, 6.v.1987, 1♀; Karlova Pláň-Karlovec (distr. Bruntál), sweeping undergrowth of alder forest, 27.v.1987, 1♀; Karlova Pláň-Volárenský potok (distr. Bruntál), sweeping undergrowth of alder forest, 20.vii.1987, 1♀, all J. Roháček leg. (SMOC). SLOVAKIA: 15♂13♀ (JRO, SMOC, UKB), for localities see Roháček (1983, 1986, 1993, 1995, 2009, 2011).

#### Diagnosis.

Body length: male 2.06–2.46 mm, female 2.20–2.90 mm. Male: abdomen with *T5* and *S8* somewhat shorter than in *Herniosina bequaerti*; *S1+2* strongly bulging; *S5* with a pair of small, shortly digitiform processes (Fig. 36); epandrium with a long dorsolateral seta (Fig. 28); cerci long, with 2 projections (Figs 28, 30), medial blunt and half-length of lateral, lateral tapered distally, with sinuate outer margin (Fig. 30) and long curved seta; gonostylus with roundly lobate posteroventral part and with slender elongate subdorsal internal projection (Fig. 29); hypandrial rod relatively short and slender (Fig. 28); phallapodeme also short, without dorsal keel (Fig. 27); postgonite relatively long, sinuate, with apex bent medially; distiphallus with longer lateral and ventral lobes and slender funnel-shaped apex (Fig. 27). Female: postabdomen slender, with relatively narrow sclerites of 6th–8th segment (Figs 32–34); *T8* complete but medially narrowly paler-pigmented (Fig. 32); *S8* relatively large, simple, transversely suboval (Fig. 33); spermathecae pyriform with conical base (Figs 31, 35); *S10* undivided, horseshoe-shaped (Fig. 33); cerci long and slender (Figs 32–34).

#### Biology.

The species is associated with decayed herbaceous vegetation, most of known specimens having been collected from decayed leaves, hay, grass (Roháček 1978, 1983) in forests or their margins, in cold montane valleys also in open, unforested habitats; they can be also captured by soil traps in these habitats (Roháček 1980). Grundmann (1991) collected a series by Barber traps. Only occasionally it can be found in forests on excrement, rotting fungi (Roháček 1993), in runs of voles (Roháček 2009) and recently was also found in entrances of caves (Roháček 2011). Despite the latter record, the statement by Roháček (1983) that it has never been collected in caves together with *Herniosina bequaerti* remains to be correct. However, *Herniosina horrida* could possibly co-occur with *Herniosina pollex* in mouths of caves or in ravines but hitherto I cannot confirm this presupposition by records. Adults were recorded in March to August.

#### Distribution.

Only known from Central Europe (Austria, Czech Republic, Germany, Slovakia). The record from Germany (Grundmann 1991; cf. also Roháček 1993) was erroneously attributed to *Herniosina pollex* in the World catalog of Sphaeroceridae (Roháček et. al 2001).

### Herniosina pollex

Taxon classificationAnimaliaDipteraSphaeroceridae

Roháček, 1993

Figs 37–39 , 40–42 , 43–46

Herniosina pollex Roháček, 1993: 186 [both sexes, phylogenetic notes, illustr.]. Type locality: Slovakia, Slovenský kras, Stará brzotínska jaskyňa (cave); Roháček et al., 2001: 149 [catalog].

#### Type material.

Holotype ♂ labelled: „CS: Slovakia or., Slovenský kras, Stará brzotínská jask., V. Košel leg.“ (obverse of the label, handwritten), „16/87, 9.6.1987, 2-5 m“ (reverse of the label, handwritten), „Holotypus (red printed), Herniosina pollex sp. n. ♂, J. Roháček det. 1991 (handwritten)“ (label with red margin), deposited in JRO (intact). Allotype ♀ (JRO) and 11♂20♀ paratypes with the same data, deposited in JRO, PKBS, SMTD (several with genit. prep.). For data of other paratypes (20♂63♀, deposited in JRO, PKBS, SMOC) from the Czech Republic and Slovakia see Roháček (1993).

#### Other material examined.

6♂4♀ – CZECH REPUBLIC: 3♂2♀ (FSBC, MBP), for localities see Roháček (1996, 1999), Roháček & Barták (2001). RUSSIA: C. Caucasus, Kabardino-Balkariya, Nalchik env., canyon of upper course of Nalchik River, Omega-12 Cave, soil traps, 30.vii.1998–25.vi.1999, 2♂, A. G. Koval leg. (SMOC). SLOVAKIA: 1♂1♀ (SMOC), for localities see Roháček (2009).

#### Diagnosis.

Smaller species, body length: male 1.90–2.18 mm, female 2.10–2.86 mm. Male: abdomen with *T5* and *S8* distinctly shorter than in *Herniosina bequaerti*; *S1+2* protruding but with bulge reduced (Fig. 41); *S5* with a pair of small, shortly digitiform processes (Fig. 42); epandrium with dorsolateral seta distinct (Fig. 37); male cerci large, each with 2 divergent projections (Figs 37, 38), medial robust, long, digitiform and projecting posteroventrally, lateral also long but terminally slender, having 1 extremely long curved seta inserted in short lateral process (Fig. 37); gonostylus with similarly (although shortly) lobate posteroventral part as that of *Herniosina horrida* but with subdorsal internal projection keel-like and anteroventral seta much shorter (Fig. 39); hypandrial rod very long and (particularly basally) slender (Fig. 37); phallapodeme very long and with dorsal keel (Fig. 40); postgonite relatively long, straighter than in other species and with apex simply pointed (Fig. 40); distiphallus with longer lateral and ventral lobes and slender funnel-shaped apex (Fig. 40). Female: postabdomen slender, with relatively narrow sclerites of 7th–8th segment (Figs 43, 46), with *T6* relatively broad; *T8* complete but medially narrowly paler-pigmented (Fig. 43); *S8* relatively large, simple, more trapezoidal (Fig. 46) than that of *Herniosina horrida*; spermathecae shortly pyriform with conical base (Fig. 44); *S10* undivided, horseshoe-shaped (Fig. 46); cerci long and slender (Figs 43, 45, 46).

#### Biology.

Although the majority of known specimens originate from caves in Slovakia the species is not troglophilous because it occurs on decayed vegetation only in the entrance zone of caves (Roháček 1993, 2011); it can also be found on rotten wet leaves in other cold and shaded places such as ravines or narrow valleys of torrents. Hitherto, it has not been recorded from runs or burrows of mammals. In Central Europe adults were collected in March, June–August.

#### Distribution.

Known only from Central Europe (Czech Republic, Slovakia) and northern part of Central Caucasus Mts (Russia: Kabardino-Balkariya). Roháček et al. (2001) incorrectly also listed Germany but this record belongs in fact to *Herniosina horrida* (see above).

### Herniosina hamata sp. n.

Taxon classificationAnimaliaDipteraSphaeroceridae

http://zoobank.org/90E15ED5-1D90-4B01-B49C-1356186E9E61

Figs 47–48 , 49–56 , 57–65

#### Type material.

Holotype ♂ labelled: „C CYPRUS: Troodos Mts., Pedoulas env., J. Roháček leg.“, „sweeping over ruderal vegetation, 12.4.2002“, „Holotypus ♂ Herniosina hamata sp. n., J. Roháček det. 2016“ (red label). The specimen (see Fig. 47) is intact except for the left wing being detached for photography (Fig. 48) and preserved in glycerine in a coalesced plastic tube pinned below the specimen (SMOC). Paratypes: C CYPRUS: Troodos Mts, Troodos 2 km NE, 1700 m, sweeping vegetation along small creek, 11.iv.2002, 1♂8♀ (1♂1♀ genit. prep.); Troodos Mts, Troodos env., 1800 m, sweeping vegetation along spring, 11.iv.2002, 1♀ (genit. prep.); Troodos Mts, Kakopetria 2 km SW, sweeping vegetation along brook, 11.iv.2002, 1♀ (genit. prep.); Pano Platres env., Caledonia Falls, on decayed leaves by stream, 12.iv.2002, 1♂ (genit. prep.); W CYPRUS: Troodos Mts, Kykko Monastery 4 km E, sweeping undergrowth of pine forest, 12.iv.2002, 1♀, all J. Roháček leg. (SMOC). Paratypes labelled „Paratypus ♂ or ♀, Herniosina hamata sp. n., J. Roháček det. 2016“ (yellow label) in addittion to their locality labels.

#### Etymology.

The new species is named by the adjective „hamata“ to reflect its hook-like posteromedial (internal) process of the gonostylus (hamatus = Lat. provided with hooks).

#### Description.

**Male** (Fig. 47). Total body length 1.94–2.38 mm; general colour blackish brown with relatively sparse dark greyish brown microtomentum, subshining (thorax) to shining (abdomen). Head blackish brown to brown, much higher than long (Fig. 47). Frons brown anteriorly, dark brown to blackish posteriorly, sparsely microtomentose. Occiput blackish brown with microtomentum denser. Orbits, interfrontalia and ocellar triangle sparsely greyish brown microtomentose; frontal triangle poorly delimited, relatively wide, anteriorly acute and more shining than rest of frons. Cephalic chaetotaxy: *pvt* absent, only minute divergent postocellar setulae behind ocellar triangle; *occe* and *occi* subequal (or *occi* slightly longer) and about half length of *vti*; *vti* robust and longest of frontal bristles; *vte* and *oc* slightly to distinctly shorter than *vti*; 2 *ors*, posterior longer than anterior but distinctly shorter than *oc*; 3–4 relatively short *ifr*, all subequal or foremost shorter, if 4 *ifr* present, the foremost reduced to small setula; 3–6 very minute *ads* inside and below *ors*; *g* weak, hardly longer than anterior peristomal setula; *vi* as long as or longer than *vte* but thinner. Frontal lunule short and wide, brown and greyish brown microtomentose. Facial cavities below antennae dark brown, relatively shining; medial carina poorly developed but distinct. Gena brown (lightest on virbrissal angle), posteriorly dark brown, greyish brown microtomentose. Eye relatively small; its longest diameter about 2.2 times as long as smallest genal height. Antenna dark brown, relatively long; its 3rd segment distinctly tapered apically, both in lateral and dorsal view. Arista long, about 4 times as long as antenna, relatively long and densely ciliate.

*Thorax* blackish brown and dark greyish brown microtomentose, mesonotum subshining, pleuron with denser microtomentum and dull. Suturae between pleural sclerites pale brown. Scutellum large, relatively long and flat, rounded trapezoidal. Thoracic chaetotaxy: 2 *hu*, internal reduced to microseta; 2 postsutural *dc*, anterior short (only 2–3 times as long as *dc* microsetae), posterior as long as or slightly longer than basal sc; 10 dense rows of *ac* microsetae on suture; medial prescutellar ac pair prolonged, about as long as anterior dc but finer; 2 long *sc*, basal about as long as or slightly longer than scutellum, apical about 1.7 times as long as basal; 1–2 *stpl*, anterior (if present) reduced to very minute pale setula.

*Legs* brown, coxae, trochanters, knees and tarsi paler brown to ochreous. *f_2_* with a doubled row of curved but relatively short ventral setae in basal half (Fig. 54); *t_2_* ventrally with a long row of small dense spines, very reduced *va* seta and 1 distinct anteroapical seta (Fig. 54); dorsal chaetotaxy of *t_2_* as in Fig. 57, with posterodorsal seta in apical fourth long. *t_2_* : *mt_2_* = 1.85–1.89.

*Wing* (Fig. 48) with pale brown membrane and yellowish brown to dark brown veins. *C* ending at or very slightly produced beyond apex of *R_4+5_*. *R_2+3_* very slightly sinuate and also apically slightly upcurved to *C*; *R_4+5_* distinctly sinuate but its apical half nearly straight. Discal cell (*dm*) rather long, distally tapered, with small process of *M* beyond *dm-cu*; posterior outer corner of *dm* cell varies from angular with a remnant of *CuA_1_* to rounded and lacking the latter. *A_1_* slightly sinuate; anal lobe large, well developed; alula narrow, apically rounded. Wing measurements: length 2.06–2.24 mm, width 0.85–0-91 mm, *C-index* = 0.97–1.03, *rm\dm-cu* : *dm-cu* = 3.64–4.50. Haltere with yellowish brown to ochreous yellow stem and dark brown knob.

*Abdomen* blackish brown dorsally, brown ventrally. Preabdominal terga large, glabrous and shining because of reduced and sparse greyish microtomentum (restricted to *T1+2* and bases of *T3–T5*), sparsely setose but with more setae on disc than those of relatives. *T5* enlarged but not so prolonged as in *Herniosina bequaerti*. Preabdominal sterna *S1+2*–*S4* (Fig. 51) also shining but distinctly more setose than in all congeners. *S1+2* with ventral bulge reduced (Fig. 47, arrow) but somewhat protruding in the middle; *S3* and *S4* (Fig. 51) anteromedially emarginate and with characteristic lateral dark pigmentation. *S5* (Fig. 65) very shortened, reduced to a transverse strip-like and largely weakly sclerotized and pale-pigmented sclerite with only partly darker and setose lateral parts (with 5–6 setae on each side) and posteromedially provided with a pair of small digitiform processes arising from a common base. *S6+7* and *S8* asymmetrical on left side of down-curved postabdomen, most similar to those of *Herniosina pollex*, thus the latter relatively short.

*Genitalia*. Epandrium (Figs 49, 50) of medium length but comparatively broad (wider than high), with a series of longer and stronger setae mainly lateroventrally (posterior longest); also laterodorsally with 1 longer seta. Anal fissure relatively small, suboval, slightly wider than in *Herniosina pollex*. Cerci fused with epandrium, each projecting ventrally in single (medial process absent) long, digitiform, terminally somewhat dilated process with blunt apex, basally carrying 1 long seta (Figs 49, 50). Medandrium fused with cerci medially and connected by long internal arms with gonostyli (Fig. 50). Hypandrium with long anteromedial rod-like apodeme, more robust than that of *Herniosina pollex*. Gonostylus (Figs 49, 50, 56) dissimilar to those of all congeners, with pointed anteroventral corner and long anterior seta as in most relatives but ventrally externally with rounded and setose lobe and posteroventrally with distinctive, robust, dark, hook-like internal process. Aedeagal complex (Figs 52, 53, 55). Phallapodeme large and long, laterally flattened due to very large (high) dorsal and ventral keel. Aedeagus with peculiar distiphallus, most different from those of relatives because of small lateral lobes (Fig. 53), long ventromedial, posteriorly far projecting lobe (Fig. 52) and relatively short but broad funnel-shaped apex (Fig. 53); also phallophore distinctive, with short and more robust anterior part and small, slender, pointed posterior epiphallus-like projection (Fig. 52); postgonite relatively short and robust (Fig. 55), with expanded posterior lobe proximally and distinctly bent and pointed apex distally. Ejacapodeme reduced to very minute sclerite (see Fig. 53) hidden in posterior fissure of phallophore.

**Female.** Similar to male unless mentioned otherwise below. Total body length 1.90–2.54 mm. Gena sometimes paler, reddish brown anteriorly and brown posteriorly. *f_2_* ventrally without curved setae, simply setulose; *t_2_* ventrally only finely setulose and with 1 long *va* seta; also anteroapical seta somewhat longer (Fig. 61). *t_2_* : *mt_2_* = 1.65–1.85. Wing measurements: length 1.79–2.28 mm, width 0.73–0.95 mm, *C-index* = 0.92–1.13, *rm\dm-cu* : *dm-cu* = 3.41–4.40. Preabdominal terga shorter, more transverse and becoming narrower posteriorly, similarly setose as in male. Preabdominal sterna unmodified, simple, sparsely and shortly setose. *S1+2* smallest and dark pigmented only posteriorly (one fourth to half); *S3–S5* becoming wider posteriorly, *S3* and *S4* trapezoidal (wider posteriorly); *S5* transversely sub-oblong, wider but distinctly shorter than *S4*; all these sclerites dark brown and shining.

*Postabdomen* (Figs 58, 62, 63) telescopically retractible but broader than in relatives, particularly as regards 7th and 8th segments. *T6* wide and short, transversely oblong, paler brown than T7 (Fig. 58); *T7* hardly narrower than *T6* but reaching farther onto lateral side (Fig. 63), sparsely setose only at posterior margin. *T8* medially not only depigmented but distinctly divided in two dark sclerites (Fig. 58). *T10* subtriangular, distinctly wider than long (shorter than that of *Herniosina pollex*), pale-pigmented only in anterior half and dorsally with a pair of longer setae arising far each from other (Fig. 58). *S6* slightly wider, paler and more densely setulose than *S7*, shorter and more transverse (Fig. 62) than in all relatives. *S7* dark-pigmented except for small anteromedial area (Fig. 62) and with 4 longer and several short setae at posterior margin. *S8* (Figs 62, 64) narrow, of highly distinctive, posteriorly widened shape, having large subcircular to ovoid membranous (sometimes posteriorly open) window in posterior half and only 4–8 fine setulae at posterior margin. *S10* reduced to short, broadly V-shaped microtomentose and setose sclerite (Fig. 62). Spermathecae 2+1 (Figs 59, 60) blackish brown, bulbous (onion-shaped), without robust basal conical parts; terminal parts of ducts very slender and pale-pigmented. Cerci (Fig. 58, 63) distinctly wider and shorter than in all relatives (including *Herniosina pollex*), with 1 dorsal preapical and 1 apical seta long sinuate as in congeners but the latter inserted somewhat subapically (see Fig. 63).

#### Discussion.

*Herniosina hamata* sp. n. is a distinctive species, markedly different from all other species of the genus in the structures of the male and female terminalia. It is characterized by an interesting combination of plesiomorphic (e.g. reduced bulge on male *S1+2*; more setose male preabdominal sclerites; male *S5* with a pair small medial projections; male cercus projecting in single process; gonostylus without dorsal internal projection; ejacapodeme minute but present; spermathecae simple, bulbous, without conical basal part) and distinctly derived autapomorphic features (gonostylus with hook-like posteroventral internal process; phallapodeme dorsoventrally dilated by both dorsal and ventral keel; distiphallus with long posteriorly projecting medial lobe; female postabdominal segments widened; female *T8* dorsomedially divided; female *S8* with „window“; cercus robust and with subapically inserted apical seta). *Herniosina hamata* sp. n. most resembles *Herniosina pollex* but the shared characters proved to be all plesiomorphic (male *S1+2* with bulge reduced; male *S5* with a pair of small medial projections; shorter male *T5* and *S8*) and do not demonstrate their sister-species relationships. Indeed, the set of plesiomorphies of *Herniosina hamata* sp. n. indicate that it could represent a sister-taxon to its four remaining congeners (see discussion of intrageneric relationships below).

#### Biology.

**Figures 66–67.**
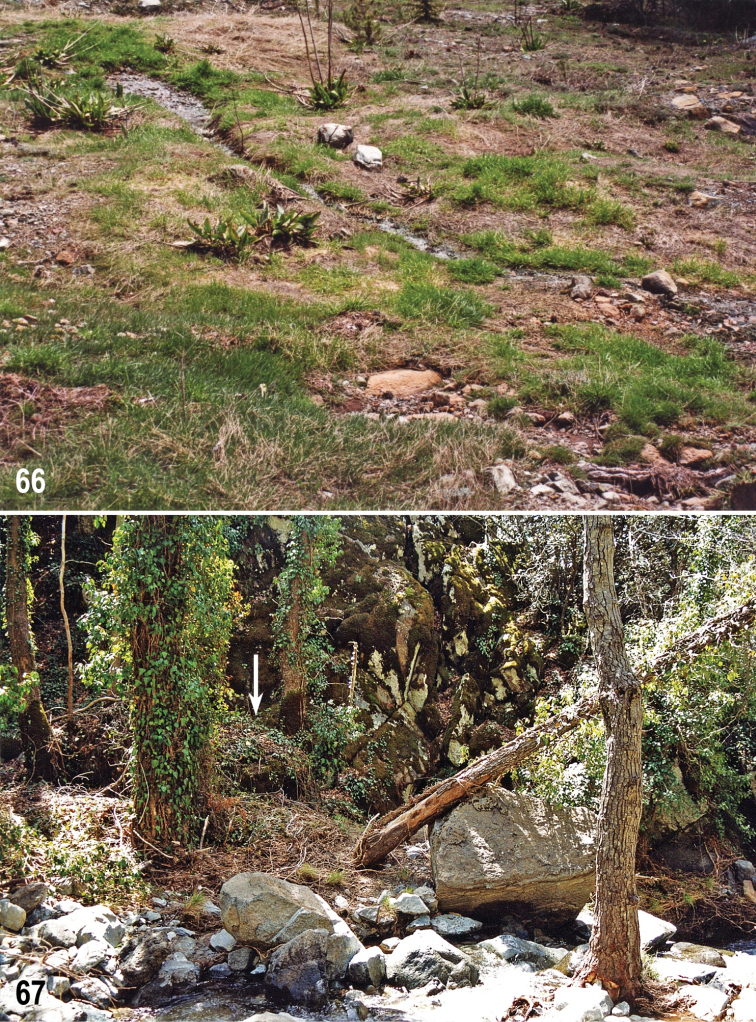
Habitats of *Herniosina hamata* sp. n. (Cyprus). **66** Shores of a small creek with decayed remnants of vegetation and shooting plants (Troodos 2 km NE, 1700 m) **67** Decayed leaves (arrow) in valley of a montane torrent below Caledonia Falls (nr. Pano Platres). Photographs by J. Roháček.

Almost all type specimens were swept from low (and sparse) vegetation growing on layers of wet rotten plant debris. This is also true for the holotype being netted from „ruderal“ vegetation on a pile of decayed leaves in a shaded ditch by a road. The longest series (9 specimens) was taken by netting over shooting plants on the wet shores of a small creek covered with decayed remnants of vegetation shortly after the snow melted (Fig. 66); also the habitat with decayed leaves close to a montane stream (Fig. 67) near the Caledonia Falls can be considered typical for the species. The altitudes of localities (all in Troodos Mts) range from about 900 m (Kakopetria env) to 1800 m (Troodos, close to Olympos Mt.) and all specimens were collected on 11–12 April.

#### Distribution.

Hitherto only known from Cyprus (Troodos Mts); first recorded as unnamed species of *Herniosina* by Roháček (2004).

### Key to identification of the species of *Herniosina*

**Table:** 

1	Male	**2**
–	Female (that of *Herniosina erymantha* unknown)	**6**
2(1)	*S1+2* with a strong protruding bulge (Figs 1, 2, 15, 17)	**3**
–	*S1+2* only slightly protruding (Figs 41, 47, 51)	**5**
3(2)	*S5* with a single long medial process that is apically forked (Figs 2, 11, 15, 19); gonostylus in lateral view sub-oblong (Fig. 23), at most ventrally emarginate (Fig. 3); phallapodeme and hypandrial rod very long (Figs 4, 20); medial process of cercus small, with apex more acute (Figs 5, 21); funnel-shaped apex of distiphallus more robust and postgonite with apex simple (Figs 6, 24, 26)	**4**
–	*S5* with 2 small digitiform medial processes (Fig. 36); gonostylus in lateral view with large posteroventral lobe (Figs 28, 29); both phallapodeme and hypandrial rod short (Fig. 28); medial process of cercus robust, with apex bluntly rounded (Figs 28, 30); funnel-shaped apex of distiphallus slender and postgonite with apex curved medially (Fig. 27)	***Herniosina horrida* (Roháček, 1978)**
4(3)	*S5* with medial process longer, in lateral view sinuous (Fig. 2), elongately conical and apically shortly forked (Fig. 11); *T5* and *S8* longer (Fig. 2); gonostylus ventrally emarginate (Fig. 3); epandrium with 1 long dorsolateral seta in addition to ventrolateral robust setae (Figs 4, 5); cercus with both processes relatively short and apically pointed (Figs 4, 5); distiphallus with ventral and lateral lobes short, also postgonite short and robust (Fig. 6)	***Herniosina bequaerti* (Villeneuve, 1917)**
–	*S5* with medial process shorter, in lateral view pestle-shaped (Fig. 15), flattened and apically deeply forked (Fig. 19); *T5* and *S8* shorter (Fig. 15); gonostylus ventrally rounded (Fig. 23); epandrium without longer dorsolateral seta (Fig. 21); cercus with only medial process short and somewhat pointed; its lateral process very long and robust, apically dilated in lateral view (Figs 20, 21); distiphallus with ventral and lateral lobes long (Fig. 26) and postgonite longer, slender, with bent but blunt apex (Fig. 24)	***Herniosina erymantha* sp. n.**
5(2)	Preabdominal sterna sparsely setose (Fig. 41); cercus with medial process very long, robust, digitiform and projecting posteroventrally; its lateral process distally slender and laterally provided with a robust long seta arising on small lobe (Figs 37, 38); gonostylus with lobe-like posteroventral part and internally with a small keel-like process (Fig. 39); phallophore anteriorly slender, ventromedial lobe of distiphallus simple (unmodified) and postgonite rather straight, with simple apex (Fig. 40)	***Herniosina pollex* Roháček, 1993**
–	Preabdominal sterna more densely setose (Fig. 51); cercus without medial process and its lateral process long, slender, apically somewhat dilated, with long seta arising more basally (Figs 49, 50); gonostylus with a robust posterior internal hook-like process directed ventrally and is posteroventral lobe smaller, knob-like (Figs 49, 50, 56); phallophore anteriorly thicker, ventromedial lobe of distiphallus projecting far posteriorly and of unusual shape (Fig. 52) and postgonite proximally dilated and with curved apex (Fig. 55)	***Herniosina hamata* sp. n.**
6(1)	*T6*, *T7*, *S6* and *S7* shorter and more transverse (Figs 58, 62); *T8* dorsomedially interrupted into 2 lateral sclerites (Figs 58, 63); *S8* with membranous window in posterior half (Figs 62, 64); spermathecae bulbous, without separate basal conical part (Figs 59, 60); cercus shorter and more robust (Fig. 58), with terminal seta inserted rather supapically (Fig. 63)	***Herniosina hamata* sp. n.**
–	*T6*, *T7*, *S6* and *S7* longer, narrower, less transverse (Figs 7, 8, 43, 46); *T8* dorsomedially complete (Fig. 7), at most with narrow, pale-pigmented, stripe (Figs 32, 43); *S8* entirely sclerotized and pigmented (Figs 8, 33, 46); spermathecae pyriform, with distinct basal conical part (Figs 10, 31, 44); cercus longer and slender (Figs 7, 32, 43), with terminal seta inserted on apex (Figs 9, 34, 45)	**7**
7(6)	*T6* narrow, about as wide as T7 (Fig. 7); *S10* divided into 2 small lateral sclerites (Figs 8, 9); *S8* small and usually with a small sclerotization in front of it in posteromedial emargination of *S7* (Fig. 8)	***Herniosina bequaerti* (Villeneuve, 1917)**
–	*T6* broad, distinctly wider than *T7* (Figs 32, 43); *S10* undivided, horseshoe-shaped (Figs 33, 46); *S8* larger, simple, without additional sclerotization (Figs 33, 46)	**8**
8(7)	*T10* longer, elongately triangular (Fig. 32); *S8* transversely suboval, with only 1 pair of setae (Fig. 33); cercus longer (Fig. 34)	***Herniosina horrida* (Roháček, 1978**)
–	*T10* shorter, transversely triangular (Fig. 43); *S8* more trapezoidal and with 1 pair of longer plus 1–2 pairs of short setae (Fig. 46); cercus shorter (Fig. 45)	***Herniosina pollex* Roháček, 1993**

## General discussion

**Relationships.** The genus *Herniosina*, as redefined here (i. e. without the Nearctic species *Herniosina voluminosa* Marshall, 1987), is a compact group of habitually very similar species differing mainly by the structures of the male and female terminalia. It is affiliated to the *Limosina* group of genera (Roháček 1982) and *Apteromyia* Vimmer, 1929 is considered its most closely allied genus (see also Marshall and Roháček 1982) based on the following synapomorphic characters: epandrium with a series of robust ventral lateral setae; distiphallus with unpaired ventromedial lobe projecting posteriorly; phallophore anteriorly slender and elongately projecting, movably attached to dorsal side of distiphallus; male cerci modified to compact processes below anal fissure.

The relationships of species within the genus *Herniosina* can be hypothetized as follows. The set of plesiomorphies of *Herniosina hamata* sp. n. (see in discussion under that species) indicate that it could represent a sister-taxon to the four remaining congeners which seem to form a monophyletic group supported by 5 synapomorphies: male preabdominal sclerites with setosity reduced; male cercus modified to 2 (lateral and medial) processes; gonostylus with dorsal internal projection; ejacapodeme absent; spermathecae pyriform, with distinct conical basal part. *Herniosina pollex*, having the male *S1+2* with bulge reduced (a plesiomorphy shared with *Herniosina hamata*) is obviously the sister-group to a cluster formed by *Herniosina horrida*, *Herniosina erymantha* sp. n. and *Herniosina bequaerti* which possess the male *S1+2* strongly protruding (bulging); moreover, this group also shares the very slender (in *Herniosina bequaerti* secondarily shortened) dorsal internal projection of the gonostylus. Both these characters can be considered synapomorphies supporting relationships of these three species. Finally, *Herniosina horrida*, with male *S5* bearing a pair small posteromedial projections (a plesiomorphy shared with *Herniosina hamata* sp. n. and *Herniosina pollex*) can be postulated as sister-group to the remaining pair, *Herniosina erymantha* sp. n. and *Herniosina bequaerti*. Relationship of these sister-species is based on 3 synapomorphies: male *S5* with posteromedial projections fused and prolonged to form a single, distally forked, process; medial apically pointed process of cercus small; funnel-shaped apex of distiphallus short and robust.

**Habitat.** *Herniosina* species seem to be originally terricolous phytosaprophagous flies associated with layers of decaying vegetation (leaf litter of broad-leaved trees in particular) in humid woodland habitats, as now known for *Herniosina hamata* sp. n., *Herniosina horrida*, *Herniosina erymantha*, and partly also *Herniosina pollex*. The latter species preferably lives on plant remnants in caves (but only close to their entrances) while *Herniosina bequaerti* became entirely adapted to cavernicolous habitats developing in various rotting matter of plant and animal origin in caves (e.g. Czižek 1916; Duda 1918, 1938) including their aphotic parts (Papp and Plachter 1976), cellars or mine galleries (see Roháček 1978, 1983) and burrows, runs and nests of various small mammals (Duda 1918; Richards 1930; Roháček 1978, 1983; Rotheray 1991). Consequently, *Herniosina pollex*, living in caves only temporarily as a component of the parietal fauna due to convenient conditions, can be classified only as a hemitroglophilous species while *Herniosina bequaerti*, having the ability to develop deep in caves in complete darkness for generations (see Papp and Plachter 1976), is considered troglophilous despite the fact it can also develop in small subterranean habitats (burrows of mammals) or in cellars (Roháček 2014). The living habits of *Herniosina* species have partly reflected on their morphology, e.g. the somewhat reduced eyes, elongate arista, relatively strong sclerotization of body. They also are poor flyers, moving usually only by running and skipping on the substrate; hence they can be caught by sweeping with difficulty and only from very low vegetation or by netting over accumulated decayed plant remnants on the ground.

**Biogeography.** Two new species described above essentially contributed to the knowledge of the distribution of *Herniosina*. Particularly, its presence in the Eastern Mediterranean was confirmed; previously there were a few records of unidentified *Herniosina* spp. from Israel (Papp and Roháček 1988: Mt. Hermon) and from Cyprus (Roháček 2004: Troodos Mts), but those of the latter are now attributed to *Herniosina hamata* sp. n. Based on available data the distribution of the genus *Herniosina* ranges from Spain in the west to Russia (Kabardino-Balkariya) in the east and from Iceland and Fennoscandia in the north to Spain, Cyprus and Israel in the south (Roháček et al. 2001; Marshall et al. 2011). The most widespread species seems to be *Herniosina bequaerti* being recorded from most of Europe (including Iceland) except for its southeastern parts (the absence of this species in caves of Balkan peninsula, cf. Séguy 1963, is particularly peculiar) and, surprisingly, *Herniosina pollex* found besides Central Europe (Czech Republic, Slovakia) unexpectedly also in the Russian Caucasus (Kabardino-Balkariya). Other species may have a more restricted distribution, viz. *Herniosina horrida* (Central Europe), *Herniosina erymantha* (Greece: Pelopponese), *Herniosina hamata* (Cyprus), Herniosina sp. cf. horrida (Israel). However, also these species can be more widely distributed considering the fact that the southern areas of W. Palaearctic are underinvestigated and that the terricolous or cavernicolous *Hernisiona* species are difficult to collect.

## Supplementary Material

XML Treatment for Herniosina

XML Treatment for Herniosina bequaerti

XML Treatment for Herniosina erymantha

XML Treatment for Herniosina horrida

XML Treatment for Herniosina pollex

XML Treatment for Herniosina hamata
